# Adaptive Event-Triggered Security Control for Nonlinear CPSs Under Coexisting FDI Attacks and Actuator Faults

**DOI:** 10.3390/s26154844

**Published:** 2026-08-01

**Authors:** Li Zhao, Wei Li, Nani Han

**Affiliations:** 1 College of Intelligent Manufacturing, Longdong University, Qingyang 745000, China; 2 College of Electrical and Information Engineering, Lanzhou University of Technology, Lanzhou 730050, China

**Keywords:** cyber–physical systems (CPSs), adaptive discrete event-triggered communication scheme (ADETCS), false data injection (FDI) attacks, actuator faults, integrated security control

## Abstract

This study addresses an integrated security control and communication co-design problem for nonlinear CPSs subject to coexisting FDI attacks and actuator faults. A novel adaptive discrete event-triggered communication scheme (ADETCS) is proposed. Its triggering threshold adapts to the system state. State estimation, fault estimation, and attack detection are all migrated to the control unit. Based on this framework, a closed-loop T–S fuzzy model is established for active defense against actuator faults and dual-end FDI attacks. A robust augmented observer is then developed via Lyapunov stability theory to jointly estimate system states, actuator faults, and FDI attacks. Sufficient conditions are further derived for an integrated security controller that unifies attack tolerance and fault tolerance. Simulation results on a quadruple-tank system show that the proposed method effectively counteracts coexisting attacks and faults while significantly reducing resource consumption. Over an 800-s horizon, data transmissions drop to 712 (8.9% transmission rate). The sensor-node computational load is also reduced from 8000 time-triggered executions to 712 event-triggered ones.

## 1. Introduction

Cyber–physical systems (CPSs) enable closed-loop regulation of physical processes through tight integration of computational and physical components [1,2,3]. As an industrial embodiment of CPSs, industrial cyber–physical systems (ICPSs) are now deployed in critical domains, including smart grids, intelligent transportation, and process industries, where failures can incur severe safety and economic consequences [4,5,6]. However, strong subsystem coupling, complex operational constraints, and external disturbances challenge the safety guarantees of conventional centralized control architectures. This motivates coordinated advances in both communication mechanisms and controller design.

Fault-tolerant control is essential for reliable ICPS operation. Actuators—the ultimate physical execution link—are prone to performance degradation or abrupt failure under harsh conditions [7,8]. The prevailing paradigm begins with passive fault-tolerant control, wherein the control law is designed a priori to tolerate prescribed faults without real-time fault information. Representative advances include event-triggered passive schemes for networked control systems [9], sliding-mode-based Lyapunov redesign approaches [10], and passive strategies ensuring perfect tracking despite additive actuator faults [11]. However, these passive methods inevitably introduce conservatism that degrades nominal performance [9,10,11]. To alleviate this limitation, active fault-tolerant control has gained prominence, wherein a fault diagnosis module first detects and isolates faults in real time [12], followed by control law reconfiguration to compensate for the identified fault. Notable results include active attitude control for flexible spacecraft [13], distributed fault-tolerant schemes for large-scale systems based on active fault diagnosis [14], and neural-network-based adaptive decentralized compensation strategies [15]. Despite these advances [9,10,11,12,13,14,15], existing studies mostly assume a trustworthy network environment and isolated fault scenarios. The coupled impact of malicious cyber attacks on fault diagnosis and fault-tolerant control loops thus remains largely unaddressed.

ICPS relies on open communication networks, dismantling the physical isolation of traditional systems and exposing them to malicious intrusions [16]. False data injection (FDI) attacks exploit coordinated deception to silently corrupt sensor measurements or actuator commands, directly threatening the estimation integrity and control validity. Adversarial capabilities have been progressively refined using learning-based dynamic attack strategies [17], stealthy sequence designs under operational constraints [18], and optimal attacks targeting distributed estimation [19]. In response to these escalating threats, research has shifted toward attack-tolerant strategies. Barboni et al. [20] exploited spatial redundancy to uncover covert attacks via distributed model-based detection. Ghaderi et al. [21] blended model-based observers with learning-based components to actively detect and neutralize FDI attacks in real-time. At the control layer, Murguia et al. [22] established security metrics and synthesis frameworks for the adversarial tolerance. Nevertheless, these mechanisms predominantly address either detection or resilient control in isolation [20,21,22]. A unified framework that simultaneously accommodates stealthy FDI attacks, actuator faults, and their coupled interactions is still lacking.

The explosive growth of sensing data in ICPS is increasingly taxing the limited network bandwidth. Although the discrete event-triggered communication scheme (DETCS) alleviates congestion through on-demand transmission, its fixed threshold cannot reflect the real-time operating states of the system, leading to redundant transmissions [23,24,25,26]. The adaptive variant (ADETCS) addresses this limitation by introducing a state-dependent dynamic threshold that enables the online regulation of communication frequency and conserves additional resources [27,28,29]. However, deeply integrating ADETCS with active intrusion tolerance and fault tolerance remains challenging. Dynamic triggering induces non-uniform sampling, which translates into time-varying transmission delays, substantially increasing the conservatism of the controller design. Meanwhile, co-locating observers and controllers on resource-constrained nodes requires departing from the traditional separate sense–compute–act architecture to achieve cross-domain load optimization. Consequently, the joint optimization of communication and computational resources while guaranteeing security performance has become a pressing bottleneck.

Table 1 summarizes representative related works and compares their features with the proposed framework.

Table 1 reveals that existing works have separately addressed communication efficiency, attack resilience, or fault tolerance; however, no unified framework simultaneously integrates adaptive event-triggered communication with active dual-end intrusion tolerance and fault tolerance. Motivated by this gap, this study focuses on a nonlinear CPS subject to coexisting FDI attacks and actuator faults, aiming to resolve the security–performance–resource trade-off.

To tackle these challenges, this study proposes a co-design framework that unifies integrated security control with adaptive event-triggered communication for nonlinear CPS subject to coexisting FDI attacks and actuator faults. The main contributions are as follows:

(1) A novel ADETCS mechanism is proposed. A dynamic triggering threshold is designed to adapt to the system trajectory, suppressing redundant data transmission at the source. This significantly improves network bandwidth utilization while preserving control performance.
(2) An active defense architecture against dual-end FDI attacks and actuator faults is constructed. The estimation tasks of system states, actuator faults, and FDI attacks are uniformly migrated to the control unit. This enables lightweight sensing terminals and achieves optimal reallocation of computational resources on the control side. Furthermore, an integrated security defense strategy combining active attack tolerance and fault tolerance is established for simultaneous mitigation of sensor- and actuator-side FDI attacks and actuator faults.
(3) A robust augmented observer and an integrated security controller are designed. Using Lyapunov stability theory, time-delay system analysis, and less-conservative inequality techniques, the existence of a robust augmented observer is established. This observer is capable of simultaneously estimating system states, actuator faults, and FDI attacks. Furthermore, integrated design conditions for an active intrusion-tolerant and fault-tolerant controller are derived based on the T–S fuzzy model.

The remainder of this paper is organized as follows. Section 2 presents the problem formulation, including the integrated security control framework, the adaptive event-triggered communication mechanism, and time-delay analysis. Section 3 is devoted to robust observer design, wherein an augmented error system is constructed, and observer gains are derived using Lyapunov–Krasovskii stability theory and LMIs. Section 4 addresses the integrated security controller design and establishes co-design conditions for the controller and event-triggering parameters. Section 5 validates the effectiveness of the proposed framework through numerical simulations on a benchmark quadruple-tank process. Finally, Section 6 concludes the paper and discusses future research directions.

**Notation:** The main symbols used throughout this study are summarized in Table 2 for quick reference.

## 2. Problem Formulation

### 2.1. Integrated Security Control Framework

**Figure 1:**
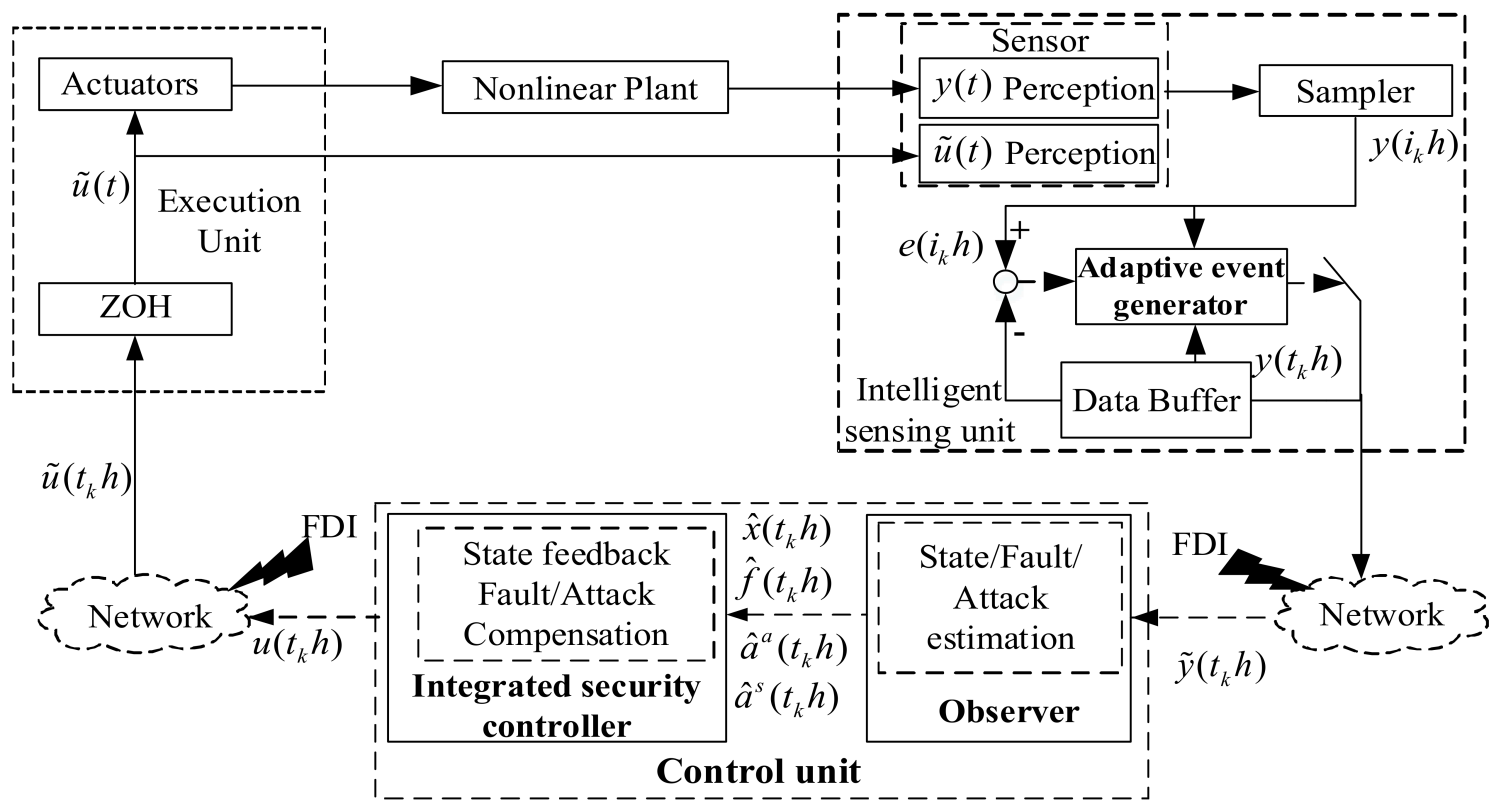
Integrated security control framework for nonlinear CPS based on ADETCS.

This section constructs an adaptive event-triggered integrated security control architecture for nonlinear CPS under FDI attacks, as illustrated in Figure 1. The design objectives are threefold: to actively defend against dual-end FDI attacks and actuator faults, to optimize computational-resource allocation across system components, and to reconcile communication economy with high operational efficiency.

As depicted in Figure 1, the proposed framework comprises five principal modules: a nonlinear plant under control, an intelligent sensing unit (including the sensor, sampler, and adaptive event generator), an actuation unit (including the zero-order hold and actuator), a control unit (housing the estimator and integrated security controller), and the communication network. The key innovations of this design are as follows. A novel adaptive event-triggering condition is devised within the intelligent sensing unit. This reduces the burden on constrained network resources by suppressing unnecessary data transmissions. It thereby achieves a more favorable trade-off between security performance and communication-aware co-design in nonlinear CPS. Furthermore, relocating the estimator from the intelligent sensing unit to the control unit offers two advantages. First, it substantially reduces the onboard data-processing burden of the sensing node. Second, it exploits the superior computational capacity of the control unit. This architectural reconfiguration also enables simultaneous deployment of active attack-tolerant strategies against dual-end FDI attacks. The overall defensive resilience of the system is thereby reinforced.

The nonlinear plant subject to concurrent FDI attacks and actuator faults is governed by the following T-S fuzzy dynamics:

(1) $$\left\{\begin{array}{l} \dot{x}(t)=\sum_{i=1}^{r}\xi_{i}(\theta (t))\left\{A_{i}x(t)+B_{i}\tilde{u}(t)+E_{fi}f(t)+E_{wi}w(t)\right\}\\ y(i_{k}h)=\sum_{i=1}^{r}\xi_{i}(\theta (t))\left\{C_{i}x(i_{k}h)+E_{vi}v(i_{k}h)\right\} \end{array}\right.$$

where $x(t)\in R^{n}$ denotes the system state; $\tilde{u}(t)\in R^{m}$ is the control input compromised by FDI attacks on the actuator-side network; $w(t)\in R^{n_{w}}$ and $v(i_{k}h)\in R^{n_{v}}$ represent the exogenous disturbance and measurement noise, respectively; $y(i_{k}h)\in R^{p}$ is the sampled measurement output; $\left\{i_{k}h,k=0,1,2,\cdots \right\}$ denotes the sequence of sampling instants; and $f(t)\in R^{n_{f}}$ corresponds to the continuously time-varying actuator fault, which is assumed to satisfy a bounded-derivative condition, namely ${\left\|\dot{f}(t)\right\|}_{2}\leq f_{1}$, where ${\left\|\cdot \right\|}_{2}$ denotes the $L_{2}$-norm of a vector. The matrices $A_{i}$, $B_{i}$ and $E_{fi}$, $E_{wi}$, $C_{i}$, $E_{vi}$ are known real matrices of appropriate dimensions.

### 2.2. Design of an Adaptive Discrete Event-Triggered Communication Scheme

In conventional DETCS, a sampled datum is transmitted only when the discrepancy between the current value and the last transmitted value exceeds a fixed threshold. Although the incorporation of an event-triggering mechanism can conserve network resources to a certain extent without unduly compromising system performance, an improperly chosen fixed threshold may engender two notable drawbacks. First, when the system operates in a steady state, an excessive number of data packets may still be transmitted, whereas in practical engineering contexts, data transmission should be curtailed once the system has attained stability with satisfactory performance. Second, prolonged intervals without triggering events can increase communication delays, which in turn introduces conservatism into the subsequent controller synthesis. The advent of adaptive discrete event-triggered communication schemes (ADETCS) offers a viable remedy for these limitations. Inspired by Ref. [27], we therefore propose a novel ADETCS wherein the triggering threshold is dynamically adjusted in accordance with the real-time operational behavior of the system. The overarching objective is to achieve a more favorable trade-off between system performance and communication resource economy.

The next data transmission instant is defined as

(2) $$t_{k+1}h=t_{k}h+\underset{l\in \mathbb{N}}{\min}\left\{lh|e^{T}(i_{k}h)\Phi e(i_{k}h)\;\leq \sigma (t_{k}h){\hat{x}}^{T}(t_{k}h)\Phi \hat{x}(t_{k}h)\right\}$$

where Φ is a positive-definite weighting matrix; $e(i_{k}h)=\hat{x}(i_{k}h)-\hat{x}(t_{k}h)$ denotes the state estimation error; $\hat{x}(i_{k}h)$ is the estimated system state at the current instant; and $\hat{x}(t_{k}h)$ is the estimated state at the most recent triggering instant. Here, $i_{k}h=t_{k}h+lh$ with l∈ℕ, $i_{k}h\in (t_{k},t_{k+1}]$, and $t_{k}(k=0,1,2,\ldots )$, the sampling period is denoted by h. Moreover, the sequence of triggering instants satisfies $\left\{t_{0},t_{1},t_{2},\ldots \right\}\subset \left\{0,1,2,\ldots \right\}$. As dictated by Equation (2), the transmission decision depends jointly on the estimation error $e(i_{k}h)$, the latest triggered state estimate $\hat{x}(t_{k}h)$, and the triggering parameter $\sigma (t_{k}h)$. The latter, $\sigma (t_{k}h)$, evolves according to the following adaptive law:

(3) $$\left\{\begin{array}{l} \sigma (t_{k}h)=\min\left\{\max\left[\sigma_{m},\lambda \sigma (t_{k-1}h\right],\sigma_{M}\right\}\\ \lambda =1-\frac{2\alpha }{\pi }\mathrm{atan}(\Delta -\varepsilon )\\ \Delta =\beta \left(\frac{\left\|\hat{x}(t_{k}h)\right\|-\left\|\hat{x}(t_{k-1}h)\right\|}{\left\|\hat{x}(t_{k}h)\right\|}\right) \end{array}\right.$$

where $\sigma_{m}>0$, $\sigma_{m}$ and $\sigma_{M}$ denote the lower and upper bounds of $\sigma (t_{k}h)$, respectively; $\sigma (0)=\sigma_{m}$, and α≥0, β≥0, ε>0 are given constants.

**Remark** **1.** *Unlike the fixed triggering parameter* σ *in Ref. [23], the adaptive triggering parameter in (2) is denoted as* $\sigma (t_{k}h)$*. It varies dynamically with the system state according to (3). Compared with a static threshold, this dynamic adjustment mechanism facilitates more efficient utilization of network communication resources.*

**Remark** **2.** *In this study, the arctangent function* atan(⋅)*, combined with parameters* α *and* β*, is utilized to automatically adjust the threshold parameter* $\sigma (t_{k}h)$*, where* $\mathrm{atan}(\cdot )\in \left[-\frac{\pi }{2},\frac{\pi }{2}\right]$*. Specifically, if* Δ−ε>0*, it follows that* 0<λ<1*, leading to* $\sigma (t_{k+1}h)<\sigma (t_{k}h)$*. Notably, if* α=0 *or* β=0*, the proposed ADETCS (3) degenerates into a fixed-threshold event-triggered mechanism [23]. Consequently, the proposed ADETCS can be regarded as a generalized form of the fixed event-triggered mechanism. Furthermore, in contrast to the work in Ref. [27], an upper bound*  $\sigma_{M}$ *is imposed on the triggering threshold. This aims to constrain the transmission delay when the system reaches a steady state, thereby preventing scenarios where data transmission is halted for extended periods during stability. Additionally, a small positive constant* ε>0 *is introduced to fine-tune the output of the* atan(⋅) *function.*

**Remark** **3.** *The adaptive threshold* $\sigma (t_{k+1}h)$ *is designed to resolve the intrinsic trade-off between control performance and communication resource consumption. A smaller threshold tightens the triggering condition, thereby increasing the transmission frequency and improving system performance through more frequent state feedback; conversely, a larger threshold relaxes the condition to suppress transmissions and conserve bandwidth, at the cost of coarser feedback updates during steady-state regulation. Importantly, the adaptive law dynamically contracts the threshold under large errors to restore performance margins, and expands it during quiescent phases to avoid redundant traffic. Consequently, the transmission rate is regulated in real time according to actual control demands, while closed-loop stability and the prescribed disturbance-rejection performance remain guaranteed regardless of the threshold magnitude. This achieves a performance-aware balance between control quality and communication efficiency.*

### 2.3. Time-Delay Analysis of CPS Under ADETCS

From the adaptive event-triggered condition (2), it is evident that data transmission screened by the ADETCS occurs in a non-uniform manner. The transmission interval is denoted as $\left[t_{k}h,t_{k+1}h\right)$, and the corresponding transmission period is defined as $T_{k}=t_{k+1}h-t_{k}h$. Although the controlled plant described in this work is continuous, the computations performed by the observer in the intelligent sensing unit and the controller in the control unit are implemented digitally. Furthermore, the introduction of ADETCS results in the non-uniform transmission of data that satisfies the system requirements. To address this typical sampled-data system, time-delay system theory is employed for analysis [30]. This approach transforms the non-uniform transmission characteristics into time delays, thereby allowing the design methods for the corresponding observer and controller to be discussed within a continuous framework.

The time-delay function is defined as follows:

(4) $$\tau_{1}(t)=t-t_{k}h,\;t\in [t_{k}h,t_{k+1}h)$$

By the definition of the time-delay function (4) and the principles of the adaptive event-triggered mechanism, this function satisfies $0\leq \tau_{1}(t)<h_{1}$, where $h_{1}$ is the upper bound of the time-delay function. Additionally, $\dot{\tau }(t)=1$. In this non-uniformly sampled data system, the update of the control input $\tilde{u}(t)$ is implemented using a zero-order hold (ZOH). The holding interval of the ZOH is synchronized with the non-uniform transmission intervals.

## 3. ADETCS-Based Robust Observer Design

### 3.1. Construction of the Augmented Error System

When FDI attacks are injected into the communication networks on both sides, the system can be described as follows:

(5) $$\left\{\begin{array}{l} \tilde{u}(t_{k}h)=u(t_{k}h)+{Ν}_{a}a^{a}(t_{k}h)\\ \tilde{y}(t_{k}h)=y(t_{k}h)+{Ν}_{s}a^{s}(t_{k}h)\; \end{array}\right.$$

where $a^{a}(t_{k}h)$ and $a^{s}(t_{k}h)\;$ denote the non-uniform sampled values of the continuously time-varying actuator-side and sensor-side network FDI attacks $a^{a}(t)$ and $a^{s}(t)$, respectively. $u(t_{k}h)$ represents the control signal calculated by the controller, while $\tilde{u}(t_{k}h)$ denotes the actual control input subjected to the actuator-side network FDI attack. $y(t_{k}h)$ is the sampled measurement output received by the control unit in the absence of sensor-side network attacks, whereas $\tilde{y}(t_{k}h)$ represents the actual sampled measurement output received by the control unit after being subjected to the sensor-side network FDI attack. Additionally, $N_{a}$ and $N_{s}$ are attack weighting matrices of appropriate dimensions. Consistent with the continuous time-varying faults, it is assumed here that the continuously time-varying FDI attacks satisfy the bounded derivative norm condition: ${\left\|{\dot{a}}^{a}(t)\right\|}_{2}\leq a$, ${\left\|{\dot{a}}^{s}(t)\right\|}_{2}\leq s$, where ${\left\|\cdot \right\|}_{2}$ denotes the $L_{2}$ norm of a vector.

Based on the sampled measurement output $\tilde{y}(t_{k}h)$ received by the control unit, a zero-order hold (ZOH) embedded within the control unit is first utilized to maintain the signal. The holding interval of the ZOH is $\left[t_{k}h,t_{k+1}h\right)$.

By introducing the state transformation $x(t)=e^{-\alpha_{1}t}\zeta (t)$, Equations (1) and (5) can be transformed as follows:

(6) $$\left\{\begin{array}{l} \dot{\zeta }(t)=\sum_{i=1}^{r}\xi_{i}(\theta (t))\left[{\tilde{A}}_{i}\zeta (t)\right.+{Β}_{i}u_{\alpha_{1}}(t)+{\tilde{E}}_{1}{\tilde{f}}_{\alpha_{1}}(t)+\left.E_{wi}w_{\alpha_{1}}(t)\right]\\ {\tilde{y}}_{\alpha_{1}}(t)=\sum_{i=1}^{r}\xi_{i}(\theta (t))\left[C_{i}\zeta (t-\tau_{1}(t))\right.+{\tilde{E}}_{2}{\tilde{f}}_{\alpha_{1}}(t)+\left.E_{vi}v_{\alpha_{1}}(t-\tau_{1}(t))\right] \end{array}\right.$$

where ${\tilde{A}}_{i}=A_{i}+\alpha_{1}I$, $u_{\alpha_{1}}(t)=e^{\alpha_{1}t}u(t)$, $w_{\alpha_{1}}(t)=e^{\alpha_{1}t}w(t)$, $y_{\alpha_{1}}(t)=e^{\alpha_{1}(t-\tau_{1}(t))}y(t)$, $v_{\alpha_{1}}(t-\tau_{1}(t))=$$e^{\alpha_{1}(t-\tau_{1}(t))}v(t-\tau_{1}(t))$, ${\tilde{f}}_{\alpha_{1}}(t)={\left[\begin{array}{ccc} e^{\alpha_{1}t}f(t) & e^{\alpha_{1}t}a^{aT}(t-\tau_{1}(t) & e^{\alpha_{1}t}a^{sT}(t-\tau_{1}(t) \end{array}\right]}^{T}$, $t\in [t_{k}h,t_{k+1}h)$, For notational convenience, ${\tilde{f}}_{\alpha_{1}}(t)$ is referred to as the augmented fault vector. The matrices ${\tilde{E}}_{1}$ and ${\tilde{E}}_{2}$ are defined as: ${\tilde{E}}_{1}=\left[\begin{array}{ccc} E_{fi} & B_{i}N_{a} & 0 \end{array}\right]$, ${\tilde{E}}_{2}=$$\left[\begin{array}{ccc} 0 & 0 & N_{s} \end{array}\right]$.

Furthermore, by introducing the state transformation $\hat{x}(t)=e^{-\alpha_{1}t}\hat{\zeta }(t)$, a robust $H_{\infty }$ estimator for state and fault estimation with α-stability is obtained as follows:

(7) $$\left\{\begin{array}{l} \dot{\hat{\zeta }}(t)=\sum_{i=1}^{r}\sum_{j=1}^{r}\xi_{i}(\theta (t))\xi_{j}(\theta (t))\left\{{\tilde{A}}_{i}\hat{\zeta }(t)\right.+B_{i}u_{\alpha_{1}}(t)+{\tilde{E}}_{1}{\hat{\tilde{f}}}_{\alpha_{1}}(t)-L_{j}e^{\alpha_{1}\tau_{1}(t)}\left.\left[{\hat{\tilde{y}}}_{\alpha_{1}}(t)-{\tilde{y}}_{\alpha_{1}}(t)\right]\right\}\\ {\hat{\tilde{y}}}_{\alpha_{1}}(t)=\sum_{i=1}^{r}\xi_{i}(\theta (t))\left\{C_{i}\right.\left.\hat{\zeta }(t-\tau_{1}(t))+{\tilde{E}}_{2}{\hat{\tilde{f}}}_{\alpha_{1}}(t-\tau_{1}(t))\right\}\\ {\dot{\hat{\tilde{f}}}}_{\alpha_{1}}(t)=\sum_{i=1}^{r}\xi_{j}(\theta (t))\left\{-e^{\alpha_{1}\tau_{1}(t)}F_{j}\left[{\hat{\tilde{y}}}_{\alpha_{1}}(t)-{\tilde{y}}_{\alpha_{1}}(t)\right]\right\} \end{array}\right.$$

where $L_{j}$ and $F_{j}$ are the gain matrices for state and augmented fault estimation to be designed. $\hat{\zeta }(t)$, ${\hat{\tilde{y}}}_{\alpha_{1}}(t)$ and ${\hat{\tilde{f}}}_{\alpha_{1}}(t)$ represent the estimated system state, the observer output, and the estimated fault, respectively.

Defining the error variables as $e_{x\alpha_{1}}(t)=\hat{\zeta }(t)-\zeta (t)$, $e_{y\alpha_{1}}(t)={\hat{\tilde{y}}}_{\alpha_{1}}(t)-{\tilde{y}}_{\alpha_{1}}(t)$, $e_{f\alpha_{1}}(t)=$${\hat{\tilde{f}}}_{\alpha_{1}}(t)-{\tilde{f}}_{\alpha_{1}}(t)$ the error system is derived as:

(8) $$\left\{\begin{array}{l} {\dot{e}}_{x\alpha_{1}}(t)=\sum_{i=1}^{r}\sum_{j=1}^{r}\xi_{i}(\theta (t))\xi_{j}(\theta (t))\left[{\tilde{A}}_{i}e_{x\alpha_{1}}(t)+{\tilde{E}}_{1}e_{\tilde{f}\alpha_{1}}(t)-L_{j}C_{i}e^{\alpha_{1}\tau_{1}(t)}e_{x\alpha_{1}}(t-\tau_{1}(t))\right.\\ \;-L_{j}{\tilde{E}}_{2}e^{\alpha_{1}\tau_{1}(t)}e_{\tilde{f}\alpha_{1}}(t)+L_{j}E_{vi}e^{\alpha_{1}\tau_{1}(t)}v_{\alpha_{1}}(t-\tau_{1}(t))\left.-E_{wi}w_{\alpha_{1}}(t)\right]\\ {\dot{e}}_{\tilde{f}\alpha_{1}}(t)=\sum_{i=1}^{r}\sum_{j=1}^{r}\xi_{i}(\theta (t))\xi_{j}(\theta (t))\left\{-e^{\alpha_{1}\tau_{1}(t)}\left[F_{j}C_{i}e_{x\alpha_{1}}(t-\tau_{1}(t))+F_{j}{\tilde{E}}_{2}e_{\tilde{f}\alpha_{1}}(t-\tau_{1}(t))\right.\right.\\ \;\left.-F_{j}E_{vi}v_{\alpha_{1}}(t-\tau_{1}(t))\right]\left.-{\dot{\tilde{f}}}_{\alpha_{1}}(t)\right\} \end{array}\right.$$

For convenience of analysis, we further define ${\tilde{e}}_{\alpha_{1}}(t)={\left[{e_{x\alpha_{1}}}^{T}(t)\;{e_{\tilde{f}\alpha_{1}}}^{T}(t)\right]}^{T}$. Then, based on Equation (8), the following augmented error system is obtained:

(9) $$\begin{array}{l} {\dot{\tilde{e}}}_{\alpha_{1}}(t)=\sum_{i=1}^{r}\sum_{j=1}^{r}\xi_{i}(\theta (t))\xi_{j}(\theta (t))\left[{\bar{A}}_{i}{\tilde{e}}_{\alpha_{1}}(t)-{\tilde{B}}_{i}e^{\alpha_{1}\tau_{1}(t)}{\tilde{e}}_{\alpha_{1}}(t-\tau_{1}(t))\right.\\ \;\left.-{\tilde{E}}_{wi}{\tilde{w}}_{\alpha_{1}}(t)+{\tilde{L}}_{j}E_{vi}e^{\alpha_{1}\tau_{1}(t)}v_{\alpha_{1}}(t-\tau_{1}(t))\right]\; \end{array}$$

where ${\bar{A}}_{i}=\left[\begin{array}{cc} {\tilde{A}}_{i} & {\tilde{E}}_{1}\\ 0 & 0 \end{array}\right],\;{\tilde{B}}_{i}={\tilde{L}}_{j}{\tilde{C}}_{i},\;{\tilde{L}}_{j}=\left[\begin{array}{c} L_{j}\\ F_{j} \end{array}\right],\;{\tilde{C}}_{i}=\left[\begin{array}{cc} C_{i} & {\tilde{E}}_{2} \end{array}\right],\;{\tilde{E}}_{wi}=\left[\begin{array}{cc} E_{wi} & 0\\ 0 & I \end{array}\right],\;{\tilde{w}}_{\alpha_{1}}(t)=\left[\begin{array}{c} w_{\alpha_{1}}(t)\\ {\dot{\tilde{f}}}_{\alpha_{1}}(t) \end{array}\right]$.

### 3.2. Design Objectives of the Robust Observer

Under the ADETCS framework, considering the nonlinear CPS subject to coexisting two-sided network FDI attacks and actuator fault, and targeting the augmented error system (9), the objective of designing a robust $H_{\infty }$ observer for state and augmented fault estimation is to determine appropriate estimator gain matrices $L_{j}$ and $F_{j}$ such that the following conditions are satisfied:

In the absence of disturbances, i.e., when ${\tilde{w}}_{\alpha_{1}}(t)=0$ and $v_{\alpha_{1}}(t-\tau_{1}(t))=0$, the augmented error system (9) is asymptotically stable, which implies $\alpha_{1}$-stability;

In the presence of disturbances, i.e., when ${\tilde{w}}_{\alpha_{1}}(t)\neq 0$ and $v_{\alpha_{1}}(t-\tau_{1}(t))\neq 0$, the augmented error system (9) satisfies the $H_{\infty }$ performance index: ${\left\|{\bar{e}}_{\alpha_{1}}(t)\right\|}_{2}^{2}\leq \gamma_{1}^{2}{\left\|{\bar{w}}_{\alpha_{1}}(t)\right\|}_{2}^{2}$$+\gamma_{2}^{2}\sum_{k=0}^{\infty }(t_{k+1}h-t_{k}h){\left\|v_{\alpha_{1}}(t_{k}h)\right\|}_{2}^{2}$, where $\gamma_{1}$ denotes the disturbance attenuation level, and ${\left\|\cdot \right\|}_{2}$ represents the $L_{2}$ norm of a vector.

### 3.3. Design Method of the Robust Observer

**Theorem** **1.** *Under the ADETCS, given positive scalars* $\gamma_{1}$*,* $\gamma_{2}$*,* $s_{1}$*,* $s_{2}$*,* $s_{3}$*,* $h_{1}$*,* $\alpha_{1}$*, for the nonlinear augmented error system (9) subject to coexisting FDI attacks and actuator fault, if there exist the symmetric positive-definite matrix* P *and matrices* X*,* $Y_{j}$ *of appropriate dimensions such that the following matrix inequalities hold:*

(10) $$\left[\begin{array}{ccccc} \Xi_{11} & \Xi_{12} & \Xi_{13} & \Xi_{14} & 0\\ {}* & \Xi_{22} & \Xi_{23} & \Xi_{24} & h_{1}s_{1}e^{2\alpha_{1}\tau_{1}(t)}{\tilde{C}}_{i}^{T}{Y_{j}}^{T}\\ {}* & * & \Xi_{33} & \Xi_{34} & 0\\ {}* & * & * & \Xi_{44} & 0\\ {}* & * & * & * & -h_{1}s_{1}e^{2\alpha_{1}\tau_{1}(t)}P \end{array}\right]<0\;$$

(11) $$\;\left[\begin{array}{ccccc} \Xi_{11}^{\left(1\right)} & \Xi_{12}^{\left(1\right)} & \Xi_{13}^{\left(1\right)} & \Xi_{14}^{\left(1\right)} & X\\ {}* & \Xi_{22}^{\left(1\right)} & \Xi_{23}^{\left(1\right)} & \Xi_{24}^{\left(1\right)} & X\\ {}* & * & \Xi_{33}^{\left(1\right)} & \Xi_{34}^{\left(1\right)} & X\\ {}* & * & * & \Xi_{44}^{\left(1\right)} & X\\ {}* & * & * & * & -\frac{15s_{1}}{23h_{1}}P \end{array}\right]<0$$

(12) $$\left[\begin{array}{ccccccc} \Xi_{11}+I & \Xi_{12} & \Xi_{13} & \Xi_{14} & \Xi_{15} & \Xi_{16} & 0\\ {}* & \Xi_{22} & \Xi_{23} & \Xi_{24} & \Xi_{25} & \Xi_{26} & h_{1}s_{1}e^{2\alpha_{1}\tau_{1}(t)}{\bar{C}}_{i}^{T}{Y_{j}}^{T}\\ {}* & * & \Xi_{33} & \Xi_{34} & 0 & 0 & 0\\ {}* & * & * & \Xi_{44} & 0 & 0 & 0\\ {}* & * & * & * & -\gamma_{1}^{2}I & \Xi_{56} & h_{1}s_{1}e^{2\alpha_{1}\tau_{1}(t)}E_{vi}^{T}{Y_{j}}^{T}\\ {}* & * & * & * & * & \Xi_{66} & 0\\ {}* & * & * & * & * & * & -h_{1}s_{1}e^{2\alpha_{1}\tau_{1}(t)}P \end{array}\right]<0\;$$

(13) $$\left[\begin{array}{ccccccc} \Xi_{11}^{\left(1\right)}+I & \Xi_{12}^{\left(1\right)} & \Xi_{13}^{\left(1\right)} & \Xi_{14}^{\left(1\right)} & \Xi_{15}^{\left(1\right)} & \Xi_{16}^{\left(1\right)} & X\\ {}* & \Xi_{22}^{\left(1\right)} & \Xi_{23}^{\left(1\right)} & \Xi_{24}^{\left(1\right)} & 0 & 0 & X\\ {}* & * & \Xi_{33}^{\left(1\right)} & \Xi_{34}^{\left(1\right)} & 0 & 0 & X\\ {}* & * & * & \Xi_{44}^{\left(1\right)} & 0 & 0 & X\\ {}* & * & * & * & -\gamma_{1}^{2}I & 0 & 0\\ {}* & * & * & * & * & -\gamma_{2}^{2}I & 0\\ {}* & * & * & * & * & * & -\frac{15s_{1}}{23h_{1}}P \end{array}\right]<0\;$$

Then the augmented error system (9) is asymptotically stable and satisfies the $H_{\infty }$ performance index as given in (14). The state observer gain matrix $L_{j}$ and the augmented fault estimation matrix $F_{j}$ can be determined by ${\tilde{L}}_{j}=\left[\begin{array}{c} L_{j}\\ F_{j} \end{array}\right]=P^{-1}Y_{j}$.

(14) $${\left\|{\tilde{e}}_{\alpha_{1}}(t)\right\|}_{2}^{2}\leq \gamma_{1}^{2}{\left\|{\tilde{w}}_{\alpha_{1}}(t)\right\|}_{2}^{2}+\gamma_{2}^{2}\sum_{k=0}^{\infty }(t_{k+1}h-t_{k}h){\left\|v_{\alpha_{1}}(t_{k}h)\right\|}_{2}^{2}$$

where $\begin{array}{l} \Xi_{11}=P{\bar{A}}_{i}+{{\bar{A}}_{i}}^{T}P-s_{2}P+h_{1}s_{2}(P{\bar{A}}_{i}+{{\bar{A}}_{i}}^{T}P)+h_{1}s_{1}{{\bar{A}}_{i}}^{T}P{\bar{A}}_{i}-3X-3X^{T},\Xi_{12}=-e^{\alpha_{1}\tau_{1}(t)}Y_{j}{\tilde{C}}_{i}+s_{2}P\\ -h_{1}s_{2}e^{\alpha_{1}\tau_{1}(t)}Y_{j}{\tilde{C}}_{i}-h_{1}s_{2}e^{\alpha_{1}\tau_{1}(t)}{{\bar{A}}_{i}}^{T}P-h_{1}s_{1}e^{\alpha_{1}\tau_{1}(t)}{{\bar{A}}_{i}}^{T}Y_{j}{\tilde{C}}_{i}+X-3X^{T},\Xi_{13}=2X-3X^{T},\Xi_{14}=6X-3X^{T},\\ \Xi_{15}=e^{\alpha_{1}\tau_{1}(t)}Y_{j}E_{vi}+h_{1}s_{2}e^{\alpha_{1}\tau_{1}(t)}Y_{j}E_{vi}+h_{1}s_{1}e^{\alpha_{1}\tau_{1}(t)}{{\bar{A}}_{i}}^{T}Y_{j}E_{vi},\Xi_{16}=-P{\tilde{E}}_{wi}-h_{1}s_{2}P{\tilde{E}}_{wi}-h_{1}s_{1}{{\bar{A}}_{i}}^{T}P{\tilde{E}}_{wi},\\ \Xi_{22}=-s_{2}P+h_{1}s_{2}e^{\alpha_{1}\tau_{1}(t)}(Y_{j}{\tilde{C}}_{i}+{{\tilde{C}}_{i}}^{T}{Y_{j}}^{T})+h_{1}s_{3}P+X+X^{T},\Xi_{23}=2X+X^{T},\Xi_{24}=6X+X^{T},\\ \Xi_{25}=-h_{1}s_{2}e^{\alpha_{1}\tau_{1}(t)}Y_{j}E_{vi},\Xi_{26}=h_{1}s_{2}e^{\alpha_{1}\tau_{1}(t)}P{\tilde{E}}_{wi}+h_{1}s_{1}e^{\alpha_{1}\tau_{1}(t)}{{\tilde{C}}_{i}}^{T}{Y_{j}}^{T}{\tilde{E}}_{wi},\Xi_{33}=2(X+X^{T}),\Xi_{34}=6X+\\ X^{T},\Xi_{44}=2(X+X^{T}),\Xi_{56}=-h_{1}s_{1}e^{\alpha_{1}\tau_{1}(t)}E_{vi}^{T}{Y_{j}}^{T}{\tilde{E}}_{wi},\Xi_{66}=-\gamma_{2}^{2}I+h_{1}s_{1}{\tilde{E}}_{wi}^{T}P{\tilde{E}}_{wi},\Xi_{11}^{\left(1\right)}=P{\bar{A}}_{i}+{{\bar{A}}_{i}}^{T}P\\ -s_{2}P-3X-3X^{T},\Xi_{12}^{\left(1\right)}=-e^{\alpha_{1}\tau_{1}(t)}Y_{j}{\tilde{C}}_{i}+s_{2}P+X-3X^{T},\Xi_{13}^{\left(1\right)}=2X-3X^{T},\Xi_{14}^{\left(1\right)}=6X-3X^{T},\\ \Xi_{15}^{\left(1\right)}=e^{\alpha_{1}\tau_{1}(t)}Y_{j}E_{vi},\Xi_{16}^{\left(1\right)}=-P{\tilde{E}}_{wi},\Xi_{22}^{\left(1\right)}=-h_{1}s_{3}P-s_{2}P+X+X^{T},\Xi_{23}^{\left(1\right)}=2X+X^{T},\Xi_{24}^{\left(1\right)}=6X+X^{T}, \end{array}$ $\Xi_{33}^{\left(1\right)}=2(X+X^{T}),\Xi_{34}^{\left(1\right)}=6X+2X^{T},\;\Xi_{44}^{\left(1\right)}=6(X+X^{T}).$

**Proof.**  Consider the following Lyapunov–Krasovskii functional:

(15) $$\begin{array}{ll} V(t)= & {\tilde{e}}_{\alpha_{1}}^{T}(t)P{\tilde{e}}_{\alpha_{1}}(t)+(h_{1}-\tau_{1}(t)){\varphi_{1}}^{T}(t)S\varphi_{1}(t)+(h_{1}-\tau_{1}(t))\tau_{1}(t){\tilde{e}}_{\alpha_{1}}^{T}(t-\\ & \tau_{1}(t))Q{\tilde{e}}_{\alpha_{1}}(t-\tau_{1}(t))+(h_{1}-\tau_{1}(t))\int_{t-\tau_{1}(t)}^{t}{\dot{\tilde{e}}}_{\alpha_{1}}^{T}(s)R{\dot{\tilde{e}}}_{\alpha_{1}}(t)ds\; \end{array}$$

where $\varphi_{1}(t)={\tilde{e}}_{\alpha_{1}}(t)-{\tilde{e}}_{\alpha_{1}}(t-t_{1}(t))$, P, Q, R, S are symmetric positive-definite matrices. □

First, consider the case when ${\tilde{w}}_{\alpha_{1}}(t)=0$ and $v_{\alpha_{1}}(t-\tau_{1}(t))=0$. The augmented error system (9) is asymptotically stable. Taking the time derivative of Equation (15) along the trajectories of the error system (9) yields:

(16) $$\begin{array}{l} \dot{V}(t)=2{\tilde{e}}_{\alpha_{1}}^{T}(t)P{\dot{\tilde{e}}}_{\alpha_{1}}(t)-{\varphi_{1}}^{T}(t)S\varphi_{1}(t)+2(h_{1}-\tau_{1}(t)){\varphi_{1}}^{T}(t)S{\dot{\tilde{e}}}_{\alpha_{1}}(t)+\\ \;2(h_{1}-\tau_{1}(t)){\tilde{e}}_{\alpha_{1}}^{T}(t-\tau_{1}(t))Q{\tilde{e}}_{\alpha_{1}}(t-\tau_{1}(t))-h_{1}{\tilde{e}}_{\alpha_{1}}^{T}(t-\tau_{1}(t))Q{\tilde{e}}_{\alpha_{1}}(t-\tau_{1}(t))-\\ \;\int_{t-\tau_{1}(t)}^{t}{\dot{\tilde{e}}}_{\alpha_{1}}^{T}(s)R\dot{\tilde{e}}(t)ds+(h_{1}-\tau_{1}(t)){\dot{\tilde{e}}}_{\alpha_{1}}^{T}(s)R{\dot{\tilde{e}}}_{\alpha_{1}}(t) \end{array}$$

To handle the integral term $\int_{t-\tau_{1}(t)}^{t}{\dot{\tilde{e}}}_{\alpha_{1}}^{T}(s)R{\dot{\tilde{e}}}_{\alpha_{1}}(t)ds$ in (16), we employ the Affine Bessel–Legendre inequality [31] and obtain:

(17) $$-\int_{t-\tau_{1}(t)}^{t}{\dot{\tilde{e}}}_{\alpha_{1}}^{T}(s)R{\dot{\tilde{e}}}_{\alpha_{1}}(t)ds\leq -{\psi_{1}}^{T}(t)\Theta \psi_{1}(t)$$

where $\psi_{1}^{T}(t)=\left[\left.\begin{array}{cccc} {\tilde{e}}_{\alpha_{1}}^{T}(t) & {\tilde{e}}_{\alpha_{1}}^{T}(t-\tau_{1}(t)) & \frac{1}{\tau_{1}(t)}\Omega_{0}^{T} & \frac{1}{\tau_{1}(t)}\Omega_{1}^{T} \end{array}\right]\right.,\Omega_{0}=\int_{t-\tau_{1}(t)}^{t}{\tilde{e}}_{\alpha_{1}}(s)ds,$ $\Omega_{1}=\int_{t-\tau_{1}(t)}^{t}\left(2\frac{s-t+\tau_{1}(t)}{\tau_{1}(t)}-1\right){\tilde{e}}_{\alpha_{1}}(s)ds,\Theta =XH_{2}+H_{2}^{T}X^{T}-\tau_{1}(t)X{\bar{R}}_{1}X^{T},$ ${\bar{R}}_{1}=\mathrm{diag}\{\begin{array}{ccc} R^{-1} & \frac{1}{3}R^{-1} & \frac{1}{5}R^{-1} \end{array}\}$, $H_{2}=\left[\begin{array}{cccc} I & -I & 0 & 0\\ I & I & -2I & 0\\ I & -I & 0 & I \end{array}\right].$

Let $M_{11}=\left[\begin{array}{cccc} I & 0 & 0 & 0 \end{array}\right],M_{12}=\left[\begin{array}{cccc} {\bar{A}}_{i} & -e^{\alpha_{1}\tau_{1}(t)}{\tilde{L}}_{j}{\tilde{C}}_{i} & 0 & 0 \end{array}\right],$ $M_{13}=\left[\begin{array}{cccc} 0 & I & 0 & 0 \end{array}\right],$ $M_{14}=\left[\begin{array}{cccc} I & -I & 0 & 0 \end{array}\right].$

Then, ${\tilde{e}}_{\alpha_{1}}(t)=M_{11}\psi_{1}(t),{\dot{\tilde{e}}}_{\alpha_{1}}(t)=M_{12}\psi_{1}(t),{\tilde{e}}_{\alpha_{1}}(t-\tau_{1}(t))$$=M_{13}\psi_{1}(t),\varphi_{1}(t)=M_{14}\psi_{1}(t).$

Thus, we obtain:

(18) $$\dot{V}(t)\leq {\psi_{1}}^{T}(t)[\Sigma_{11}+(h_{1}-\tau_{1}(t))\Sigma_{12}+\tau_{1}(t)\Sigma_{13}]\psi_{1}(t)<0$$

where $\Sigma_{11}=2M_{11}^{T}PM_{12}-M_{14}^{T}SM_{14}-h_{1}M_{13}^{T}QM_{13}-(XH_{2}+H_{2}^{T}X^{T})$, $\Sigma_{13}=X\bar{R}X^{T}$,$\Sigma_{12}=$$2M_{14}^{T}SM_{12}+2M_{13}^{T}QM_{13}+M_{12}^{T}RM_{12}$.

If the condition $\Sigma_{11}+(h_{1}-\tau_{1}(t))\Sigma_{12}+\tau_{1}(t)\Sigma_{13}<0$ holds, it follows that ${\dot{V}}_{1}(t)<0$. Consequently, the augmented error system (9) is asymptotically stable.

Furthermore, according to [32], the condition $\Sigma_{11}+(h_{1}-\tau_{1}(t))\Sigma_{12}+\tau_{1}(t)\Sigma_{13}<0$ can be equivalently expressed as:

(19) $$\Sigma_{11}+h_{1}\Sigma_{12}<0,\;\Sigma_{11}+h_{1}\Sigma_{13}<0$$

When ${\tilde{w}}_{\alpha_{1}}(t)\neq 0$ and $v_{\alpha_{1}}(t-\tau_{1}(t))\neq 0$, the augmented error system (10) satisfies the following $H_{\infty }$ performance index:

(20) $$J_{1}=\dot{V}(t)+{\tilde{e}}_{\alpha_{1}}^{T}(t){\tilde{e}}_{\alpha_{1}}(t)-(\gamma_{1}^{2}{\tilde{w}}_{\alpha_{1}}^{T}(t){\tilde{w}}_{\alpha_{1}}(t)+\gamma_{2}^{2}v_{\alpha_{1}}^{T}(t_{k}h)v_{\alpha_{1}}(t_{k}h))<0$$

Combining Equations (18) and (19), and simultaneously defining:

$${\tilde{e}}_{\alpha_{1}}(t)=M_{21}{\bar{\psi }}_{1}(t),\;{\dot{\tilde{e}}}_{\alpha_{1}}(t)=M_{22}{\bar{\psi }}_{1}(t),\;\varphi_{1}(t)=M_{24}{\bar{\psi }}_{1}(t),\;{\tilde{e}}_{\alpha_{1}}(t-\tau_{1}(t))=M_{23}{\bar{\psi }}_{1}(t),\;\psi_{1}(t)=M_{25}{\bar{\psi }}_{1}(t),\;{\left[\begin{array}{cc} v_{\alpha_{1}}^{T}(t_{k}h) & {\tilde{w}}_{\alpha_{1}}^{T}(t) \end{array}\right]}^{T}=M_{26}{\bar{\psi }}_{1}(t).$$

where ${\bar{\psi }}_{1}^{T}(t)=\left[\begin{array}{cccccc} {\tilde{e}}_{\alpha_{1}}^{T}(t) & {\tilde{e}}_{\alpha_{1}}^{T}(t-\tau_{1}(t)) & \frac{1}{\tau_{1}(t)}\Omega_{0}^{T} & \frac{1}{\tau_{1}(t)}\Omega_{1}^{T} & v_{\alpha_{1}}^{T}(t-\tau_{1}(t)) & {\tilde{w}}_{\alpha_{1}}^{T}(t) \end{array}\right]$, $M_{21}=\left[\begin{array}{cccccc} I & 0 & 0 & 0 & 0 & 0 \end{array}\right]$, $M_{22}=\left[\begin{array}{cccccc} {\bar{A}}_{i} & -e^{\alpha_{1}\tau_{1}(t)}{\tilde{L}}_{j}{\tilde{C}}_{i} & 0 & 0 & e^{\alpha_{1}\tau_{1}(t)}{\tilde{L}}_{j}E_{vi} & -{\tilde{E}}_{wi} \end{array}\right]$, $M_{23}=\left[\begin{array}{cccccc} 0 & I & 0 & 0 & 0 & 0 \end{array}\right]$, $M_{24}=\left[\begin{array}{cccccc} I & -I & 0 & 0 & 0 & 0 \end{array}\right]$, $M_{25}=\left[\begin{array}{cccccc} I & 0 & 0 & 0 & 0 & 0\\ 0 & I & 0 & 0 & 0 & 0\\ 0 & 0 & I & 0 & 0 & 0\\ 0 & 0 & 0 & I & 0 & 0 \end{array}\right]$, $M_{26}=\left[\begin{array}{cccccc} 0 & 0 & 0 & 0 & I & 0\\ 0 & 0 & 0 & 0 & 0 & I \end{array}\right]$.

Thus, we obtain:

(21) $$J_{1}\leq {{\bar{\psi }}_{1}}^{T}(t)[\Sigma_{21}+(h_{1}-\tau_{1}(t))\Sigma_{22}+\tau_{1}(t)\Sigma_{23}]{\bar{\psi }}_{1}(t)<0$$

where $\Sigma_{21}=2{M_{21}}^{T}PM_{22}-{M_{24}}^{T}SM_{24}-h_{1}{M_{23}}^{T}QM_{23}-{M_{25}}^{T}(XH_{2}+{H_{2}}^{T}X^{T})M_{25}+{M_{21}}^{T}M_{21}$$-\gamma_{1}^{2}{M_{26}}^{T}M_{26}$, $\Sigma_{22}=2{M_{24}}^{T}SM_{22}+2{M_{23}}^{T}QM_{23}.\;\Sigma_{23}={M_{25}}^{T}X\bar{R}X^{T}M_{25}$.

According to Ref. [32], Equation (21) can be equivalently expressed as:

(22) $$\Sigma_{21}+h_{1}\Sigma_{22}<0,\Sigma_{21}+h_{1}\Sigma_{23}<0$$

Due to the presence of nonlinear terms in Equations (19) and (22), these matrix inequalities cannot be directly solved using standard linear matrix inequality (LMI) solvers. To address this, a parameterization method is employed to linearize the nonlinear terms. Let $R=s_{1}P$, $S=s_{2}P$, $Q=s_{3}P$, $Y_{j}=P{\tilde{L}}_{j}$ where $s_{1}$, $s_{2}$, $s_{3}$ are tunable scalars. By expanding these expressions and applying the Schur complement lemma, Equations (10)–(13) are derived. Consequently, the robust observer gain matrices to be designed can be computed as ${\tilde{L}}_{j}=P^{-1}Y_{j}$.

Subsequently, integrating Equation (20) from 0 to +∞ yields:

(23) $$\begin{array}{ll} V(+\infty )-V(0)< & -\int_{0}^{+\infty }{\tilde{e}}_{\alpha_{1}}^{T}(t){\tilde{e}}_{\alpha_{1}}(t)dt+\gamma_{1}^{2}\int_{0}^{+\infty }{\tilde{w}}_{\alpha_{1}}^{T}(t){\tilde{w}}_{\alpha_{1}}(t)dt+\\ & \gamma_{2}^{2}\sum_{k=0}^{\infty }\left(t_{k+1}h-t_{k}h)v_{\alpha_{1}}^{T}(t_{k}h)v_{\alpha_{1}}(t_{k}h\right) \end{array}$$

From this, it follows that:

(24) $$\int_{0}^{+\infty }{\tilde{e}}_{\alpha_{1}}^{T}(t){\tilde{e}}_{\alpha_{1}}(t)dt<\gamma_{1}^{2}\int_{0}^{+\infty }{\tilde{w}}_{\alpha_{1}}^{T}(t){\tilde{w}}_{\alpha_{1}}(t)dt+\gamma_{2}^{2}\sum_{k=0}^{\infty }\left(t_{k+1}h-t_{k}h)v_{\alpha_{1}}^{T}(t_{k}h)v_{\alpha_{1}}(t_{k}h\right)$$

That is, the condition ${\left\|{\tilde{e}}_{\alpha_{1}}(t)\right\|}_{2}^{2}\leq \gamma_{1}^{2}{\left\|{\tilde{w}}_{\alpha_{1}}(t)\right\|}_{2}^{2}+\gamma_{2}^{2}\sum_{k=0}^{\infty }(t_{k+1}h-t_{k}h){\left\|v_{\alpha_{1}}(t_{k}h)\right\|}_{2}^{2}$ holds. This completes the proof of the theorem.

## 4. Design of an Integrated Security Controller

### 4.1. Modeling of the Closed-Loop Nonlinear CPS

Based on the system state estimation $\hat{x}(t_{k}h)$ and the augmented fault estimation $\hat{\tilde{f}}(t_{k}h)$ obtained in Section 3.3, where $\hat{\tilde{f}}(t_{k}h)={\left[{\hat{f}}^{T}(t_{k}h){\hat{a}}^{aT}(t_{k}h){\hat{a}}^{sT}(t_{k}h)\right]}^{T}$, the integrated secure control strategy is formulated as:

(25) $$u(t_{k}h)=\sum_{i=1}^{r}\sum_{j=1}^{r}\xi_{i}(\theta (t))\xi_{j}(\theta (t))\left[K_{j}\hat{x}(t_{k}h)-B_{j}^{+}E_{fi}\hat{f}(t_{k}h)-N_{a}{\hat{a}}^{a}(t_{k}h)\right]$$

where $K_{j}$ is the controller gain matrix to be designed, and $B_{j}^{+}$ satisfies $(I-B_{i}B_{j}^{+})E_{fi}$ =0. Additionally, $\hat{f}(t_{k}h)=[I\;0\;0]\cdot \hat{\tilde{f}}(t_{k}h)$, ${\hat{a}}^{a}(t_{k}h)=[0\;I\;0]\cdot \hat{\tilde{f}}(t_{k}h)$, ${\hat{a}}^{s}(t_{k}h)=[0\;0\;I]\cdot \hat{\tilde{f}}(t_{k}h)$ can be regarded as the decoupling of FDI attacks on the sensor-side network. Furthermore, the first term in Equation (25) represents the state feedback control strategy based on a state observer, while the latter two terms denote the active compensation for actuator faults and actuator-side network FDI attacks.

By further combining Equations (1), (6) and (25), the closed-loop model of the nonlinear CPS can be described as

(26) $$\begin{array}{l} \dot{x}(t)=\sum_{i=1}^{r}\sum_{j=1}^{r}\xi_{i}(\theta (t))\xi_{j}(\theta (t))\left[A_{i}x(t)-B_{i}K_{j}x(t-\tau_{1}(t))-B_{i}K_{j}e_{x}(t-\tau_{1}(t))\right.\\ \;-E_{fi}e_{f}(t-\tau_{1}(t))+\tau_{1}(t)E_{fi}\dot{f}(t)-B_{i}N_{a}e_{a}(t-\tau_{1}(t))+\tau_{1}(t)B_{i}N_{a}{\dot{a}}^{a}(t)\\ \;+\left.E_{wi}w(t)\right],\;t\in [t_{k}h,t_{k+1}h)\; \end{array}$$

By introducing the state transformation $x(t)=e^{-\alpha_{2}t}\zeta (t)$, the above equation can be transformed into the following closed-loop CPS model with guaranteed α-stability:

(27) $$\begin{array}{ll} \dot{\zeta }(t) & =\sum_{i=1}^{r}\sum_{j=1}^{r}\xi_{i}(\theta (t))\xi_{j}(\theta (t))\left[{\hat{A}}_{i}\zeta (t)-B_{i}K_{j}e^{\alpha_{2}\tau_{1}(t)}\zeta (t-\tau_{1}(t))-B_{i}K_{j}e^{\alpha_{2}\tau_{1}(t)}e_{x_{\alpha_{2}}}(t-\tau_{1}(t))\right.\\ & -E_{fi}e^{\alpha_{2}\tau_{1}(t)}e_{f_{\alpha_{2}}}(t-\tau_{1}(t))+\tau_{1}(t)E_{fi}{\dot{f}}_{\alpha_{2}}(t)-B_{i}N_{a}e^{\alpha_{2}\tau_{1}(t)}e_{a_{\alpha_{2}}}(t-\tau_{1}(t))+\tau_{1}(t)B_{i}N_{a}{\dot{a}}_{\alpha_{2}}^{a}(t)\\ & +\left.E_{wi}w_{\alpha_{2}}(t)\right],\;t\in [t_{k}h,t_{k+1}h)\; \end{array}$$

where ${\hat{A}}_{i}=A_{i}+\alpha_{2}I$, $w_{\alpha_{2}}(t)=e^{\alpha_{2}t}w(t)$, ${\dot{f}}_{\alpha_{2}}(t)=e^{\alpha_{2}t}\dot{f}(t)$, $e_{x_{\alpha_{2}}}(t-\tau_{1}(t))=e^{\alpha_{2}(t-\tau_{1}(t))}e_{x}(t-\tau_{1}(t))$, $e_{f_{\alpha_{2}}}(t-\tau_{1}(t))=e^{\alpha_{2}(t-\tau_{1}(t))}e_{f}(t-\tau_{1}(t))$, $e_{a_{\alpha_{2}}}(t-\tau_{1}(t))=e^{\alpha_{2}(t-\tau_{1}(t))}e_{a}(t-\tau_{1}(t))$, ${\dot{a}}_{\alpha_{2}}^{a}(t)=e^{\alpha_{2}\tau_{1}(t)}{\dot{a}}^{a}(t)$.

### 4.2. Design Objectives of the Integrated Secure Controller

Under the ADETCS, for a class of nonlinear CPS (27) subject to simultaneous dual-end network FDI attacks and actuator fault, the objective of the integrated secure control and communication co-design is to jointly determine the controller gain matrix $K_{j}$ and the event-triggering weighting matrix Φ, such that the following conditions are satisfied:

(1) In the absence of disturbances, i.e., $w_{\alpha_{2}}(t)=0$, $e_{x_{\alpha_{2}}}(t-\tau_{1}(t))=0$, $e_{f_{\alpha_{2}}}(t-\tau_{1}(t))=0$, $e_{a_{\alpha_{2}}}(t-\tau_{1}(t))=0$, the closed-loop nonlinear CPS (27) is asymptotically stable;
(2) In the presence of disturbances, i.e., $w_{\alpha_{2}}(t)\neq 0$, $e_{x_{\alpha_{2}}}(t-\tau_{1}(t))\neq 0$, $e_{f_{\alpha_{2}}}(t-\tau_{1}(t))\neq 0$, $e_{a_{\alpha_{2}}}(t-\tau_{1}(t))\neq 0$, the closed-loop nonlinear CPS (27) satisfies the $H_{\infty }$ performance index: ${\left\|\zeta (t)\right\|}_{2}^{2}\leq \gamma_{3}^{2}({\left\|w_{\alpha_{2}}(t)\right\|}_{2}^{2}+\sum_{k=0}^{\infty }\left(t_{k+1}h-t_{k}h\right)({\left\|e_{x_{\alpha_{2}}}(t_{k}h)\right\|}_{2}^{2}+{\left\|e_{f_{\alpha_{2}}}(t_{k}h)\right\|}_{2}^{2}+{\left\|e_{a_{\alpha_{2}}}(t_{k}h)\right\|}_{2}^{2}))$, where $\gamma_{3}$ denotes the disturbance attenuation level, and ${\left\|\cdot \right\|}_{2}$ represents the $L_{2}$ norm of a vector.
(3) The system possesses integrated secure defense capabilities against FDI attacks and actuator faults, while effectively conserving network resources.

### 4.3. Design Method of the Integrated Secure Controller

**Theorem** **2.** *Under the ADETCS, given the parameters* $\gamma_{3}$*,* σ*,* $n_{1}$*,* $n_{2}$*,* $n_{3}$*,* $m_{1}$*,* $m_{2}$*,* $m_{3}$*,* $m_{4}$*,* $m_{5}$*,* $m_{6}$*,* $h_{2}$*, for the nonlinear CPS (27) subject to coexisting FDI attacks and actuator faults, if there exist the symmetric positive-definite matrix* $\tilde{P}$*, and matrices* Φ*,* ${\tilde{K}}_{j}$*,* $Q_{1}$*,* $Q_{2}$*,* $Q_{3}$*,* $Q_{4}$*,* $Q_{5}$*,* $Q_{6}$*,* ${\tilde{Q}}_{1}$*,* ${\tilde{Q}}_{2}$*,* ${\tilde{Q}}_{3}$*,* ${\tilde{Q}}_{4}$*,* ${\tilde{Q}}_{5}$*,* ${\tilde{Q}}_{6}$ *of appropriate dimensions, such that the following matrix inequalities hold:*

(28) $$\left[\begin{array}{cccccc} \Xi_{11}^{\left(2\right)} & \Xi_{12}^{\left(2\right)} & \Xi_{13}^{\left(2\right)} & \Xi_{14}^{\left(2\right)} & 0 & 0\\ {}* & \Xi_{22}^{\left(2\right)} & \Xi_{23}^{\left(2\right)} & \Xi_{24}^{\left(2\right)} & \Xi_{25}^{\left(2\right)} & h_{2}n_{2}e^{2\alpha_{2}\tau_{1}(t)}{\tilde{K}}_{j}^{T}\\ {}* & * & \Xi_{33}^{\left(2\right)} & \Xi_{34}^{\left(2\right)} & 0 & 0\\ {}* & * & * & \Xi_{44}^{\left(2\right)} & 0 & 0\\ {}* & * & * & * & \Xi_{55}^{\left(2\right)} & 0\\ {}* & * & * & * & * & -h_{2}n_{2}e^{2\alpha_{2}\tau_{1}(t)}\tilde{P} \end{array}\right]<0$$

(29) $$\left[\begin{array}{cccccc} \Xi_{11}^{\left(3\right)} & \Xi_{12}^{\left(3\right)} & \Xi_{13}^{\left(3\right)} & \Xi_{14}^{\left(3\right)} & \tilde{X} & 0\\ {}* & \Xi_{22}^{\left(3\right)} & \Xi_{23}^{\left(3\right)} & \Xi_{24}^{\left(3\right)} & \tilde{X} & \Xi_{26}^{\left(3\right)}\\ {}* & * & \Xi_{33}^{\left(3\right)} & \Xi_{34}^{\left(3\right)} & \tilde{X} & 0\\ {}* & * & * & \Xi_{44}^{\left(3\right)} & \tilde{X} & 0\\ {}* & * & * & * & -\frac{15n_{2}}{23h_{2}}\tilde{P} & 0\\ {}* & * & * & * & * & \Xi_{66}^{\left(3\right)} \end{array}\right]<0$$

(30) $$\left[\begin{array}{cc} \Xi_{11}^{\left(4\right)} & \Xi_{12}^{\left(4\right)}\\ {}* & \Xi_{22}^{\left(4\right)} \end{array}\right]<0$$

(31) $$\left[\begin{array}{cc} \Xi_{11}^{\left(5\right)} & \Xi_{12}^{\left(5\right)}\\ {}* & \Xi_{22}^{\left(5\right)} \end{array}\right]<0$$

(32) $$\begin{array}{l} \left[\begin{array}{cc} Q_{2} & E_{fi}^{T}{\tilde{P}}^{T}\\ {}* & Q_{1} \end{array}\right]>0,\left[\begin{array}{cc} Q_{4} & E_{fi}^{T}\tilde{S}\\ {}* & Q_{3} \end{array}\right]>0,\left[\begin{array}{cc} Q_{6} & E_{fi}^{T}\tilde{R}\\ {}* & Q_{5} \end{array}\right]>0,\\ \left[\begin{array}{cc} {\tilde{Q}}_{2} & N_{a}^{T}{B_{i}}^{T}{\tilde{P}}^{T}\\ {}* & {\tilde{Q}}_{1} \end{array}\right]>0,\left[\begin{array}{cc} {\tilde{Q}}_{4} & N_{a}^{T}{B_{i}}^{T}\tilde{S}\\ {}* & {\tilde{Q}}_{3} \end{array}\right]>0,\left[\begin{array}{cc} {\tilde{Q}}_{6} & N_{a}^{T}{B_{i}}^{T}\tilde{R}\\ {}* & {\tilde{Q}}_{5} \end{array}\right]>0 \end{array}$$

then the closed-loop nonlinear CPS (27) is asymptotically stable and satisfies the $H_{\infty }$ performance index given by (33). The integrated secure controller gain matrix $K_{j}={\left(\tilde{P}B_{i}\right)}^{+}{\tilde{K}}_{j}$ and the event-triggering matrix Φ can be co-designed. The fault compensation matrix $B_{j}^{+}$ satisfies $(I-B_{i}B_{j}^{+})E_{fi}=0$.

(33) $${\left\|\zeta (t)\right\|}_{2}^{2}\leq \gamma_{3}^{2}({\left\|w_{\alpha_{2}}(t)\right\|}_{2}^{2}+\sum_{k=0}^{\infty }\left(t_{k+1}h-t_{k}h\right)({\left\|e_{x_{\alpha_{2}}}(t_{k}h)\right\|}_{2}^{2}+{\left\|e_{f_{\alpha_{2}}}(t_{k}h)\right\|}_{2}^{2}+{\left\|e_{a_{\alpha_{2}}}(t_{k}h)\right\|}_{2}^{2}))$$

where $\begin{array}{l} \Xi_{11}^{\left(2\right)}=\tilde{P}{\hat{A}}_{i}+{{\hat{A}}_{i}}^{T}\tilde{P}-n_{1}\tilde{P}+\frac{h_{1}^{2}}{4}(m_{2}+m_{5})\tilde{P}+\frac{h_{1}^{2}}{4}(m_{3}+m_{6}){{\hat{A}}_{i}}^{T}\tilde{P}{\hat{A}}_{i}+h_{1}n_{2}{{\hat{A}}_{i}}^{T}\tilde{P}\hat{A}+h_{1}n_{1}(\tilde{P}{\hat{A}}_{i}+{{\hat{A}}_{i}}^{T}\tilde{P})\\ -3\tilde{X}-3{\tilde{X}}^{T},\Xi_{12}^{\left(2\right)}=-e^{\alpha_{2}\tau_{1}(t)}{\tilde{K}}_{j}+n_{1}\tilde{P}-\frac{h_{1}^{2}}{4}(m_{2}+m_{5})\tilde{P}-\frac{h_{1}^{2}}{4}(m_{3}+m_{6})e^{\alpha_{2}\tau_{1}(t)}{{\hat{A}}_{i}}^{T}{\tilde{K}}_{j}-h_{1}n_{2}e^{\alpha_{2}\tau_{1}(t)}{{\hat{A}}_{i}}^{T}{\tilde{K}}_{j}\\ -h_{1}n_{1}{\tilde{K}}_{j}-h_{1}n_{1}{{\hat{A}}_{i}}^{T}\tilde{P}+\tilde{X}-3{\tilde{X}}^{T},\Xi_{13}^{\left(2\right)}=2\tilde{X}-3{\tilde{X}}^{T},\Xi_{14}^{\left(2\right)}=6\tilde{X}-3{\tilde{X}}^{T},\Xi_{22}^{\left(2\right)}=-n_{1}\tilde{P}+h_{1}n_{3}\tilde{P}+\\ \frac{h_{2}^{2}}{4}(m_{2}+m_{5})\tilde{P}+h_{1}n_{1}({\tilde{K}}_{j}+{{\tilde{K}}_{j}}^{T})+\tilde{X}+{\tilde{X}}^{T},\Xi_{23}^{\left(2\right)}=2\tilde{X}+{\tilde{X}}^{T},\Xi_{24}^{\left(2\right)}=6\tilde{X}+{\tilde{X}}^{T},\Xi_{25}^{\left(2\right)}=\\ \frac{h_{2}^{2}}{4}(m_{3}+m_{6})e^{2\alpha_{2}\tau_{1}(t)}{\tilde{K}}_{j}^{T},\Xi_{33}^{\left(2\right)}=2(\tilde{X}+{\tilde{X}}^{T}),\Xi_{34}^{\left(2\right)}=6\tilde{X}+2{\tilde{X}}^{T},\Xi_{44}^{\left(2\right)}=6(\tilde{X}+{\tilde{X}}^{T}),\Xi_{55}^{\left(2\right)}=\\ -\frac{h_{2}^{2}}{4}(m_{3}+m_{6})e^{2\alpha_{2}\tau_{1}(t)}\tilde{P},\Xi_{11}^{\left(3\right)}=\tilde{P}{\hat{A}}_{i}+{{\hat{A}}_{i}}^{T}\tilde{P}-n_{1}\tilde{P}+\frac{h_{1}^{2}}{4}(m_{2}+m_{5})\tilde{P}+\frac{h_{1}^{2}}{4}(m_{3}+m_{6}){{\hat{A}}_{i}}^{T}\tilde{P}{\hat{A}}_{i}-3\tilde{X}-\\ 3{\tilde{X}}^{T}+(m_{1}+m_{4})\tilde{P},\Xi_{12}^{\left(3\right)}=-e^{\alpha_{2}\tau_{1}(t)}{\tilde{K}}_{j}+n_{1}\tilde{P}-\frac{h_{1}^{2}}{4}(m_{2}+m_{5})\tilde{P}-\frac{h_{1}^{2}}{4}(m_{3}+m_{6})e^{\alpha_{2}\tau_{1}(t)}{{\hat{A}}_{j}}^{T}{\tilde{K}}_{j}+\tilde{X}-3{\tilde{X}}^{T},\\ \Xi_{13}^{\left(3\right)}=2\tilde{X}-3{\tilde{X}}^{T},\Xi_{14}^{\left(3\right)}=6\tilde{X}-3{\tilde{X}}^{T},\Xi_{22}^{\left(3\right)}=-n_{1}\tilde{P}+\frac{h_{2}^{2}}{4}(m_{2}+m_{5})\tilde{P}+h_{1}n_{3}\tilde{P}+h_{1}n_{1}({\tilde{K}}_{j}+{{\tilde{K}}_{j}}^{T})+\\ \tilde{X}+{\tilde{X}}^{T},\Xi_{23}^{\left(3\right)}=2\tilde{X}+{\tilde{X}}^{T},\Xi_{24}^{\left(3\right)}=6\tilde{X}+{\tilde{X}}^{T},\Xi_{26}^{\left(3\right)}=\frac{h_{2}^{2}}{4}(m_{3}+m_{6})e^{2\alpha_{2}\tau_{1}(t)}{\tilde{K}}_{j}^{T},\Xi_{33}^{\left(3\right)}=2(\tilde{X}+{\tilde{X}}^{T}),\\ \Xi_{34}^{\left(3\right)}=6\tilde{X}+2{\tilde{X}}^{T},\Xi_{44}^{\left(3\right)}=6(\tilde{X}+{\tilde{X}}^{T}),\Xi_{66}^{\left(3\right)}=-\frac{h_{2}^{2}}{4}(m_{3}+m_{6})\tilde{P},\\ \Xi_{11}^{\left(4\right)}=\left[\begin{array}{cccccc} {Ζ}_{11} & {Ζ}_{12} & {Ζ}_{13} & {Ζ}_{14} & {Ζ}_{15} & {Ζ}_{16}\\ {}* & {Ζ}_{22} & {Ζ}_{23} & {Ζ}_{24} & {Ζ}_{25} & {Ζ}_{26}\\ {}* & * & {Ζ}_{33} & {Ζ}_{34} & {Ζ}_{35} & {Ζ}_{36}\\ {}* & * & * & {Ζ}_{44} & {Ζ}_{45} & {Ζ}_{46}\\ {}* & * & * & * & -\gamma_{3}^{2}I & {Ζ}_{56}\\ {}* & * & * & * & * & {Ζ}_{66} \end{array}\right],\;\Xi_{12}^{\left(4\right)}=\left[\begin{array}{cccccc} {Ζ}_{17} & Z_{18} & 0 & 0 & 0 & 0\\ Z_{27} & Z_{28} & 0 & 0 & Z_{211} & h_{1}n_{2}e^{2\alpha_{2}\tau_{1}(t)}{{\tilde{K}}_{j}}^{T}\\ 0 & 0 & 0 & 0 & 0 & 0\\ 0 & 0 & 0 & 0 & 0 & 0\\ Z_{57} & Z_{58} & 0 & 0 & Z_{511} & h_{1}n_{2}e^{2\alpha_{2}\tau_{1}(t)}{{\tilde{K}}_{j}}^{T}\\ Z_{67} & Z_{68} & 0 & 0 & 0 & 0 \end{array}\right],\\ Z_{68}=[-\frac{h_{1}^{2}}{4}(m_{3}+m_{6})-h_{1}n_{2}]e^{\alpha_{2}\tau_{1}(t)}E_{fi}^{T}\tilde{P}E_{wi},Z_{77}=-\gamma_{3}^{2}I+[\frac{h_{1}^{2}}{4}(m_{3}+m_{6})+h_{1}n_{2}]E_{a}^{T}{B_{i}}^{T}\tilde{P}B_{i}E_{a},\\ Z_{78}=[-\frac{h_{1}^{2}}{4}(m_{3}+m_{6})-h_{1}n_{2}]e^{\alpha_{2}\tau_{1}(t)}E_{a}^{T}{B_{i}}^{T}\tilde{P}E_{wi},Z_{88}=-\gamma_{3}^{2}I+[\frac{h_{1}^{2}}{4}(m_{3}+m_{6})+h_{1}n_{2}]E_{wi}^{T}\tilde{P}E_{wi},\\ Z_{99}=\sigma_{m}\Phi ,Z_{1111}=-\frac{h_{1}^{2}}{4}(m_{3}+m_{6})\tilde{P}\\ \Xi_{11}^{\left(5\right)}=\left[\begin{array}{cccccc} Z_{11}^{\prime } & Z_{12}^{\prime } & Z_{13}^{\prime } & Z_{14}^{\prime } & Z_{15}^{\prime } & Z_{16}^{\prime }\\ {}* & Z_{22}^{\prime } & Z_{23}^{\prime } & Z_{24}^{\prime } & 0 & Z_{26}^{\prime }\\ {}* & * & Z_{33}^{\prime } & Z_{34}^{\prime } & 0 & 0\\ {}* & * & * & Z_{44}^{\prime } & 0 & 0\\ {}* & * & * & * & -\gamma_{3}^{2}I & Z_{56}^{\prime }\\ {}* & * & * & * & * & Z_{66}^{\prime } \end{array}\right],\Xi_{12}^{\left(5\right)}=\left[\begin{array}{cccccc} Z_{17}^{\prime } & Z_{18}^{\prime } & 0 & 0 & \tilde{X} & 0\\ Z_{27}^{\prime } & Z_{28}^{\prime } & 0 & 0 & \tilde{X} & Z_{212}^{\prime }\\ 0 & 0 & 0 & 0 & \tilde{X} & 0\\ 0 & 0 & 0 & 0 & \tilde{X} & 0\\ Z_{57}^{\prime } & Z_{58}^{\prime } & 0 & 0 & 0 & Z_{512}^{\prime }\\ Z_{67}^{\prime } & Z_{68}^{\prime } & 0 & 0 & 0 & 0 \end{array}\right],\\ \Xi_{22}^{\left(5\right)}=\left[\begin{array}{cccccc} Z_{77}^{\prime } & Z_{78}^{\prime } & 0 & 0 & 0 & 0\\ {}* & Z_{88}^{\prime } & 0 & 0 & 0 & 0\\ {}* & * & Z_{99}^{\prime } & 0 & 0 & 0\\ {}* & * & * & -\Phi & 0 & 0\\ {}* & * & * & * & Z_{1111}^{\prime } & 0\\ {}* & * & * & * & * & Z_{1212}^{\prime } \end{array}\right]. \end{array}$

**Proof.**  The proof of Theorem 2 follows similar lines to that of Theorem 1 and is therefore omitted for brevity. □

## 5. Simulation Results and Analysis

To validate the effectiveness and feasibility of the proposed methodology, numerical simulations are conducted using the classic quadruple-tank process—a widely recognized benchmark in industrial process control [33]. Characterized by its compact structure and rich dynamics, this system is extensively employed to simulate complex industrial scenarios, including chemical processing, water treatment, and power grid dispatch. In this study, the quadruple-tank system serves as the physical plant within the ICPS framework, interconnected with the integrated secure control unit via a shared communication network, thereby establishing a comprehensive cyber–physical co-simulation testbed.

The evaluation presented in this section proceeds in four consecutive stages: first, the system description and physical parameters are introduced in Section 5.1; second, the observer and controller gains are designed and computed in Section 5.2; third, the standalone performance of the proposed scheme is validated in Section 5.3, including state/fault estimation accuracy and the integrated security control behavior under coexisting FDI attacks and actuator faults; finally, Section 5.4 provides a systematic comparative analysis against existing representative methods in terms of control performance, communication-resource consumption, and computational overhead.

### 5.1. System Description and Parameters

The system parameters are given as follows:

$\begin{array}{l} A_{1}=\left[\begin{array}{cccc} -0.016 & 0 & 0.042 & 0\\ 0 & -0.011 & 0 & 0.033\\ 0 & 0 & -0.042 & 0\\ 0 & 0 & 0 & -0.033 \end{array}\right],A_{2}=\left[\begin{array}{cccc} -0.022 & 0 & 0.061 & 0\\ 0 & -0.018 & 0 & 0.049\\ 0 & 0 & -0.064 & 0\\ 0 & 0 & 0 & -0.049 \end{array}\right],\\ A_{3}=\left[\begin{array}{cccc} -0.031 & 0 & 0.053 & 0\\ 0 & -0.021 & 0 & 0.067\\ 0 & 0 & -0.083 & 0\\ 0 & 0 & 0 & -0.061 \end{array}\right],A_{4}=\left[\begin{array}{cccc} -0.039 & 0 & 0.106 & 0\\ 0 & -0.0276 & 0 & 0.0826\\ 0 & 0 & -0.107 & 0\\ 0 & 0 & 0 & -0.0827 \end{array}\right],\\ B_{1}=\left[\begin{array}{cc} 0.083 & 0\\ 0 & 0.063\\ 0 & 0.048\\ 0.031 & 0 \end{array}\right],B_{2}=\left[\begin{array}{cc} 0.1246 & 0\\ 0 & 0.093\\ 0 & 0.071\\ 0.045 & 0 \end{array}\right],B_{3}=\left[\begin{array}{cc} 0.165 & 0\\ 0 & 0.125\\ 0 & 0.097\\ 0.063 & 0 \end{array}\right],B_{4}=\left[\begin{array}{cc} 0.2076 & 0\\ 0 & 0.1576\\ 0 & 0.13\\ 0.0776 & 0 \end{array}\right],\\ C_{1}=diag\{\begin{array}{cccc} 0.5 & 0.5 & 0.5 & 0.5 \end{array}\},C_{2}=diag\{\begin{array}{cccc} 0.48 & 0.48 & 0.48 & 0.48 \end{array}\},\\ C_{3}=diag\{\begin{array}{cccc} 0.46 & 0.46 & 0.46 & 0.46 \end{array}\},C_{4}=diag\{\begin{array}{cccc} 0.52 & 0.52 & 0.52 & 0.52 \end{array}\},\\ E_{f1}=-{\left[\begin{array}{cccc} 0.083 & 0 & 0 & 0.031 \end{array}\right]}^{T},E_{f2}=-{\left[\begin{array}{cccc} 0.1246 & 0 & 0 & 0.0464 \end{array}\right]}^{T},\\ E_{f3}=-{\left[\begin{array}{cccc} 0.167 & 0 & 0 & 0.061 \end{array}\right]}^{T},E_{f4}=-{\left[\begin{array}{cccc} 0.2076 & 0 & 0 & 0.0774 \end{array}\right]}^{T},\\ E_{v1}={\left[\begin{array}{cccc} 0.015 & 0 & 0.015 & 0.015 \end{array}\right]}^{T},E_{v2}={\left[\begin{array}{cccc} 0.0224 & 0 & 0.0224 & 0.0224 \end{array}\right]}^{T},\\ E_{v3}={\left[\begin{array}{cccc} 0.030 & 0 & 0.025 & 0.027 \end{array}\right]}^{T},E_{v4}={\left[\begin{array}{cccc} 0.0374 & 0 & 0.031 & 0.0326 \end{array}\right]}^{T}. \end{array}$

The corresponding parameters for the system model in Equation (1) are given below: $x_{i}(i=1,2,3,4)$ denotes the liquid-level variation of the i-th tank; $y_{i}(t)$ represents the observed liquid-level variation. The tanks are fed by two pumps (actuators), with $u_{i}(t)$ denoting the voltage applied to each pump. It is assumed that the fault occurs in the first input channel. The continuous time-varying fault is described as:

$$f(t)=\left\{\begin{array}{l} 0,\;t\leq 400\\ 2+2\sin0.01\pi (t-400),\;400<t\leq 800 \end{array}\right.$$

The actuator-side and sensor-side FDI attacks are assumed to take the following forms, respectively: $a^{a}(t)=\left\{\begin{array}{l} 0,\;t\leq 100\\ 2+2\sin0.01\pi (t-100),\;100<t\leq 800 \end{array}\right.,$ $a^{s}(t)=\left\{\begin{array}{l} 0,\;t\leq 200\\ 2+2\sin0.01\pi (t-200),\;200<t\leq 800 \end{array}\right.$

Select $E_{s}={\left[\begin{array}{cccc} 1 & 1 & 1 & 1 \end{array}\right]}^{T}$, $E_{a}={\left[\begin{array}{cc} 1 & 1 \end{array}\right]}^{T}$, $\sigma_{m}=0.001$, $\sigma_{M}=0.01$, $\sigma (0)=\sigma_{m}$, α=0.01, β=1, ε=0.01 with the system initial state remaining at $x_{0}={\left[4\;4\;2\;2\right]}^{T}$, and the sampling period h=0.1s.

Based on the system parameters described above, the observer and controller gains are subsequently designed in this subsection using the stability conditions derived in Theorems 1 and 2.

### 5.2. Design of Observer and Controller Gains

#### 5.2.1. Observer Gain Design

According to Theorem 1, the simulation parameters are chosen as $\gamma_{1}=4,\;\gamma_{2}=5,s_{1}=0.1,\;s_{2}=2,s_{3}=0.01,h_{1}=0.1,\alpha_{1}=0.05$, yielding the following robust $H_{\infty }$ observer gain matrices $L_{j},F_{j}$ that guarantee α-stability:

$$\begin{array}{l} L_{1}=\left[\begin{array}{cccc} 6.3932 & 1.6781 & -2.95676 & -1.9234\\ 2.8901 & 3.0123 & -2.1890 & -2.9875\\ 3.0171 & 0.5401 & -0.0876 & -1.9891\\ 2.7678 & 0.8901 & -3.2324 & 0.9123 \end{array}\right],L_{2}=\left[\begin{array}{cccc} 5.711 & 0.9241 & -3.1962 & -2.3544\\ 3.4762 & 2.2524 & -1.8601 & -2.6789\\ 2.8929 & 0.7234 & -0.1234 & -2.3456\\ 2.7312 & 0.7986 & 3.2455 & 0.8568 \end{array}\right],\;\\ L_{3}=\left[\begin{array}{cccc} 5.9035 & 0.9573 & -3.3241 & -2.3712\\ 3.6981\; & 2.1925 & -1.7833 & -2.6741\\ 2.8198 & 0.6892 & -0.1425 & -2.4083\\ 2.7812 & 0.7624 & -3.2107 & 0.7289 \end{array}\right],L_{4}=\left[\begin{array}{cccc} 5.0852 & 0.8943 & -2.8915 & -1.9705\\ 3.0174 & 1.8736 & -1.4238 & -2.3862\\ 2.3179 & 0.6387 & -0.1198 & -2.0591\\ 2.2953 & 0.6831 & -2.7812 & 0.6827 \end{array}\right],\;\\ F_{1}=\left[\;\begin{array}{cccc} 19.1743 & -9.1024 & -37.8962 & 32.3651\\ 28.9892 & -1.6135 & 0.2147 & -29.8634\\ -0.9157 & 0.0942 & 1.4521 & 1.3782 \end{array}\right],\;\\ F_{2}=\left[\begin{array}{cccc} 18.0246 & -6.4898 & -42.3173 & 34.2157\\ 29.346 & -1.8154 & 0.8912 & -31.1176\\ -1.0329 & 0.4912 & 1.123 & 1.4649 \end{array}\right],\;\\ F_{3}=\left[\begin{array}{cccc} 16.9769 & -2.9741 & -46.5214 & 36.0528\\ 30.1289 & -3.2871 & 0.7846 & -32.3015\\ -1.1573 & 0.5846 & 1.0983 & 1.4317 \end{array}\right],\\ F_{4}=\left[\begin{array}{cccc} 14.2163 & -2.1738 & -41.8935 & 32.4176\\ 25.9834 & -3.2704 & 0.7029 & -28.1972\\ -1.1824 & 0.6321 & 1.0184 & 1.4462 \end{array}\right]. \end{array}$$

#### 5.2.2. Controller Gain Design

In Theorem 2, the simulation parameters are selected as follows: $\gamma_{3}=4,\;m_{1}=m_{2}=m_{3}=m_{4}=m_{5}=m_{6}=1,\;n_{1}=4,n_{2}=$$0.01,n_{3}=0.1,$ $\alpha_{2}=0.06,h_{2}=2.1$.

Then, the controller gain matrices $K_{j}$ and event-triggered weight matrices Φ can be obtained respectively as:

$$\begin{array}{l} \Phi =diag\left\{\begin{array}{cccc} 1.7126 & 1.7126 & 1.7126 & 1.7126 \end{array}\right\},\\ K_{1}=\left[\begin{array}{cccc} 25.4872 & 1.8219 & -0.2845 & 11.3746\\ 2.2937 & 21.9843 & 20.0312 & -0.9127 \end{array}\right],K_{2}=\left[\begin{array}{cccc} 18.5731 & 1.1428 & -0.2916 & 6.9539\\ 1.9921 & 14.5817 & 13.8812 & -0.5018 \end{array}\right],\\ K_{3}=\left[\begin{array}{cccc} 13.2486 & 0.8934 & -0.2013 & 6.3264\\ 0.9248 & 10.7691 & 9.9935 & -0.2185 \end{array}\right],K_{4}=\left[\begin{array}{cccc} 11.8235 & 1.7537 & -0.0278 & 4.1687\\ 1.7184 & 9.0924 & 8.9243 & -1.0873 \end{array}\right]. \end{array}$$

With the observer and controller gains determined, the standalone performance of the proposed ADETCS–based integrated secure control scheme is validated in the following subsection.

### 5.3. Performance Validation

#### 5.3.1. Fault and Attack Estimation

**Figure 2:**
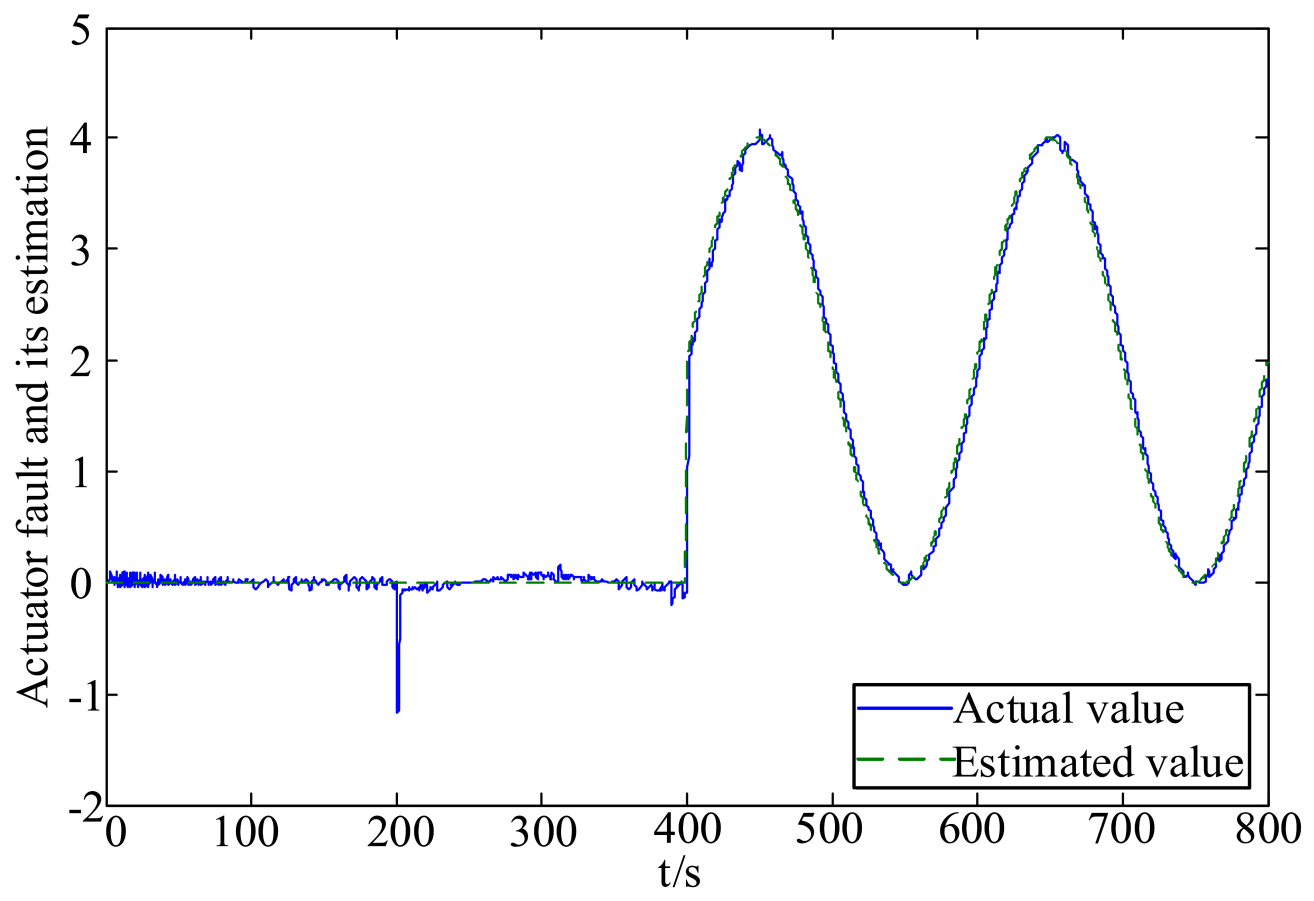
Continuous time−varying fault and its estimate.

**Figure 3:**
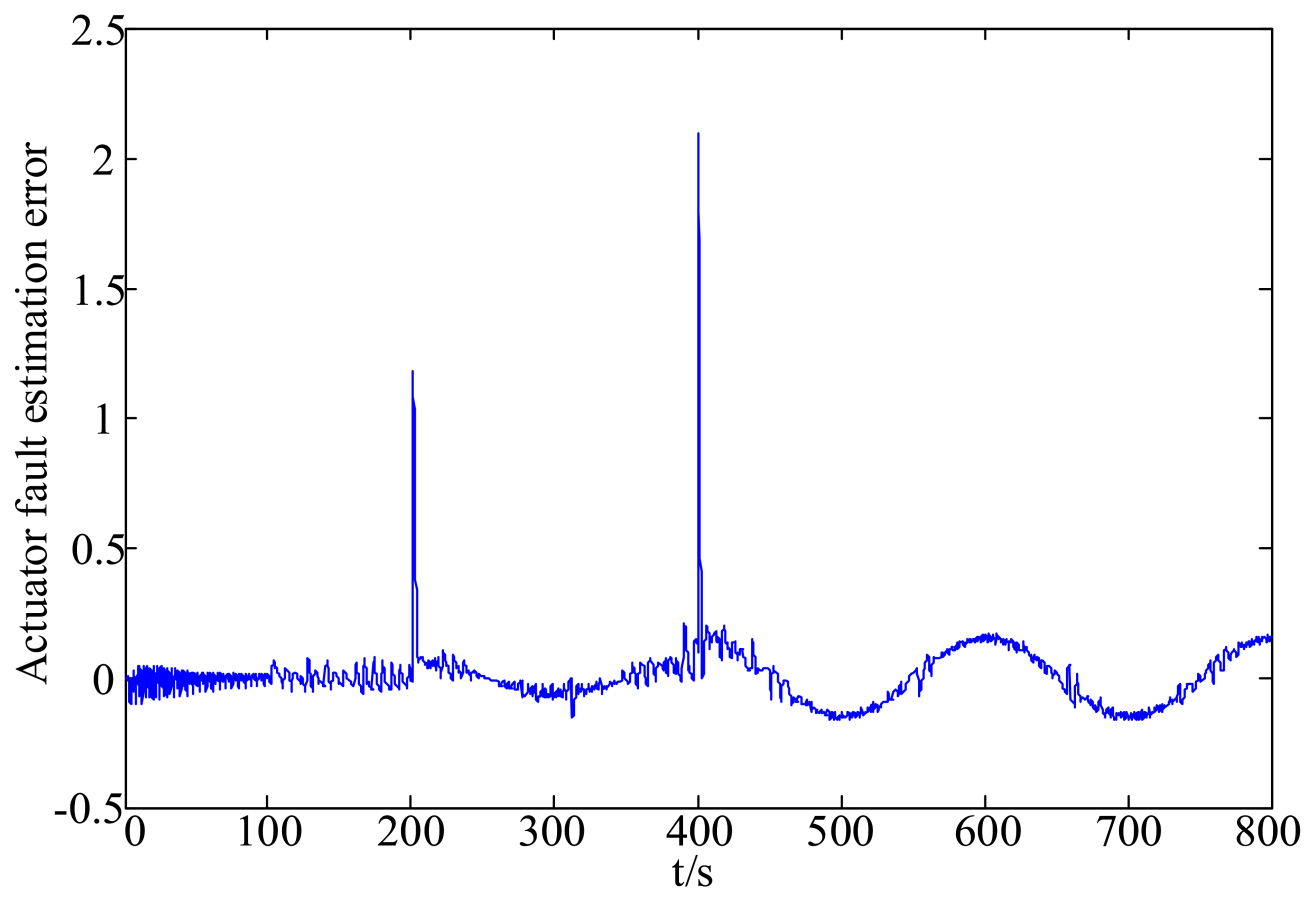
Fault estimation errors.

**Figure 4:**
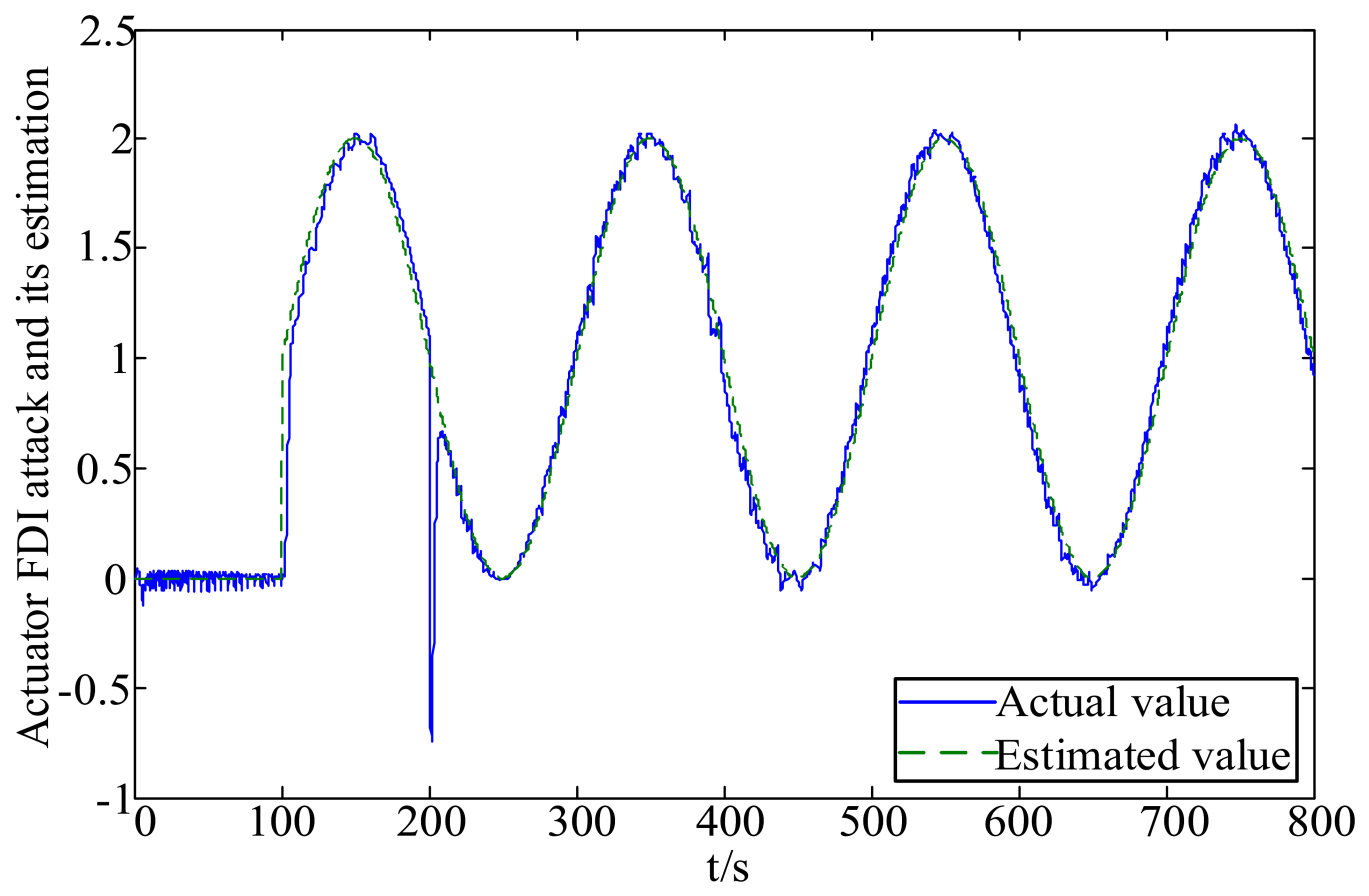
Actuator FDI attack and its estimation.

**Figure 5:**
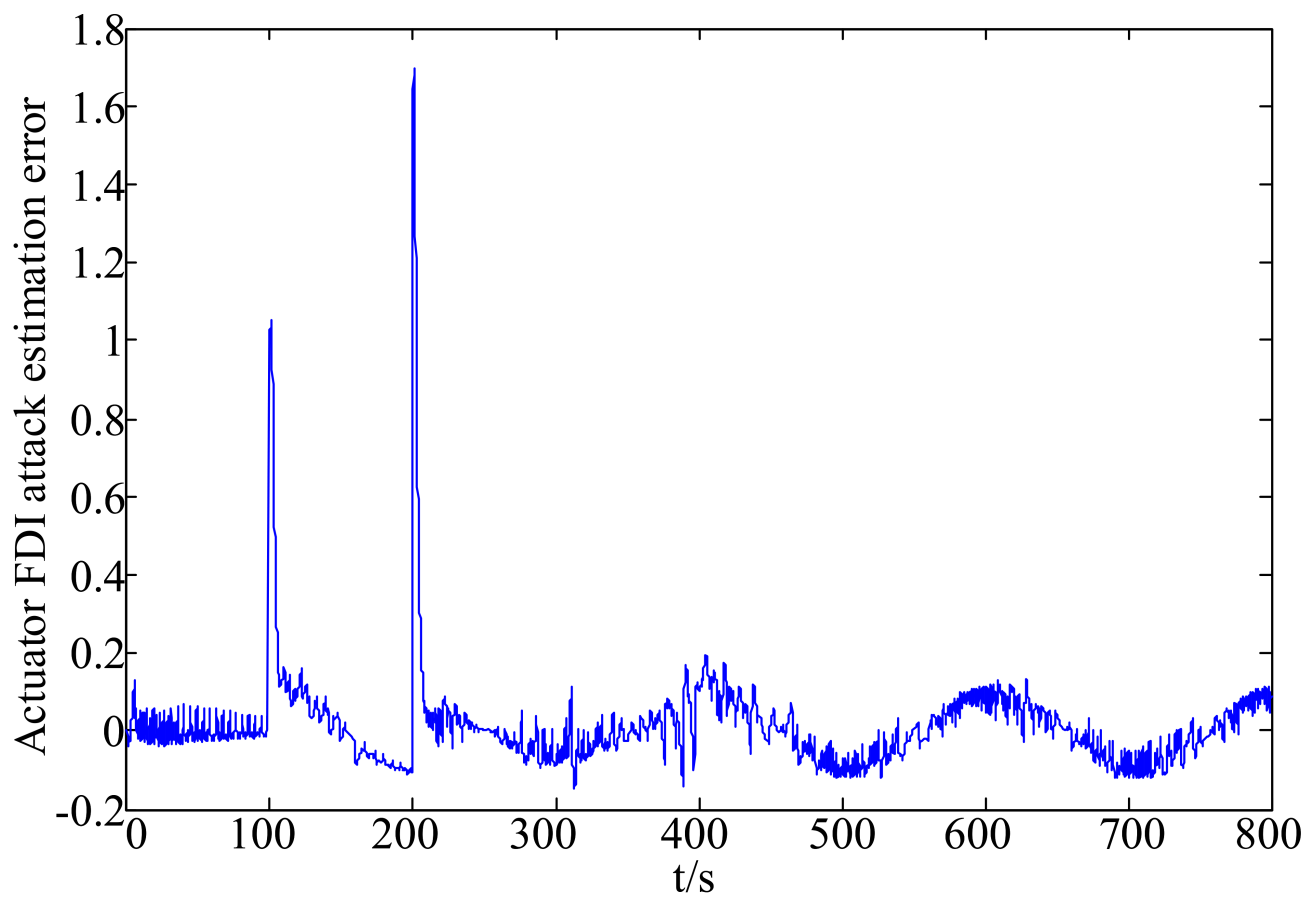
Estimation error of the actuator FDI attack.

**Figure 6:**
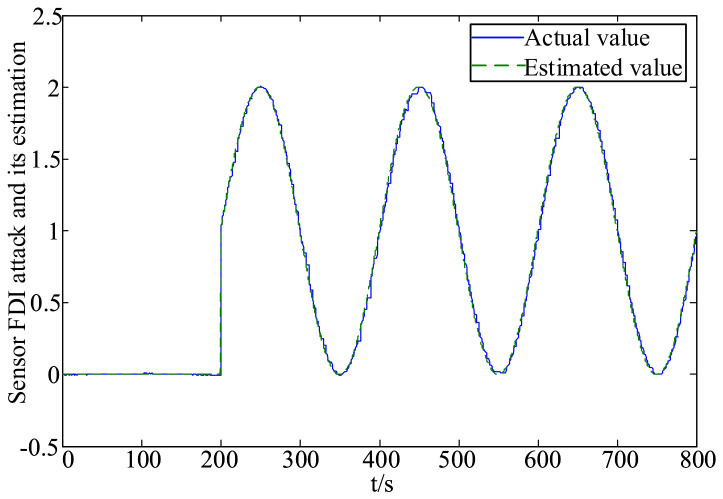
Sensor FDI attack and its estimation.

**Figure 7:**
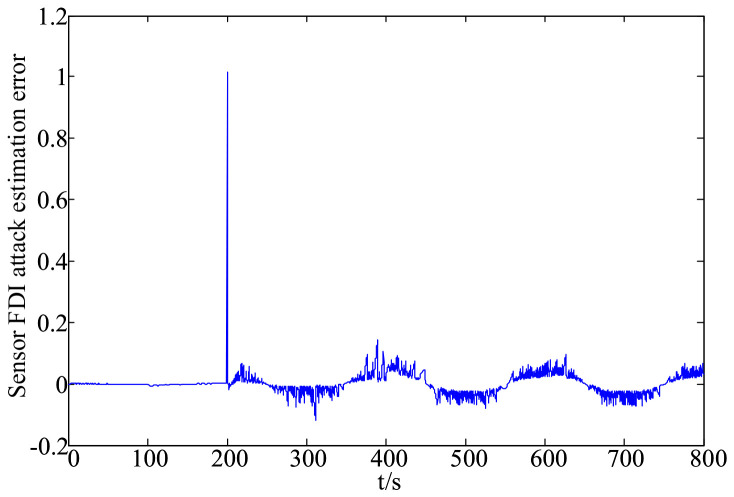
Estimation errors of the sensor FDI attack.

Under the ADETCS framework, when the system is subjected to dual-end FDI attacks and actuator faults simultaneously, the actuator fault and its estimate, the sensor-side and actuator–side FDI attacks and their estimates, together with the corresponding estimation errors, are illustrated in Figure 2, Figure 3, Figure 4, Figure 5, Figure 6 and Figure 7.

As illustrated in Figure 2, Figure 3, Figure 4, Figure 5, Figure 6 and Figure 7, when the actuator-side FDI attack, sensor-side FDI attack, and abrupt actuator fault are injected at t=100s, t=200s, and t=400s, respectively, the estimation errors exhibit brief transient excursions exclusively at the exact injection instants. Thereafter, the errors decay rapidly and remain confined within tight bounded residual sets. Specifically, the actuator fault estimation error (Figure 2 and Figure 3) is bounded within [−0.17,0.17]; the actuator-side FDI attack estimation error (Figure 4 and Figure 5) remains within [−0.15,0.15]; and the sensor-side FDI attack estimation error (Figure 6 and Figure 7) oscillates within [−0.08,0.08]. These results demonstrate that the proposed estimator achieves accurate and robust reconstruction of actuator faults and dual-end FDI attacks.

Having verified the estimation performance of the observer, we next evaluate the closed-loop system performance under the integrated security controller when confronted with coexisting FDI attacks and actuator faults.

#### 5.3.2. Integrated Security Control Under FDI Attacks and Actuator Fault

**Figure 8:**
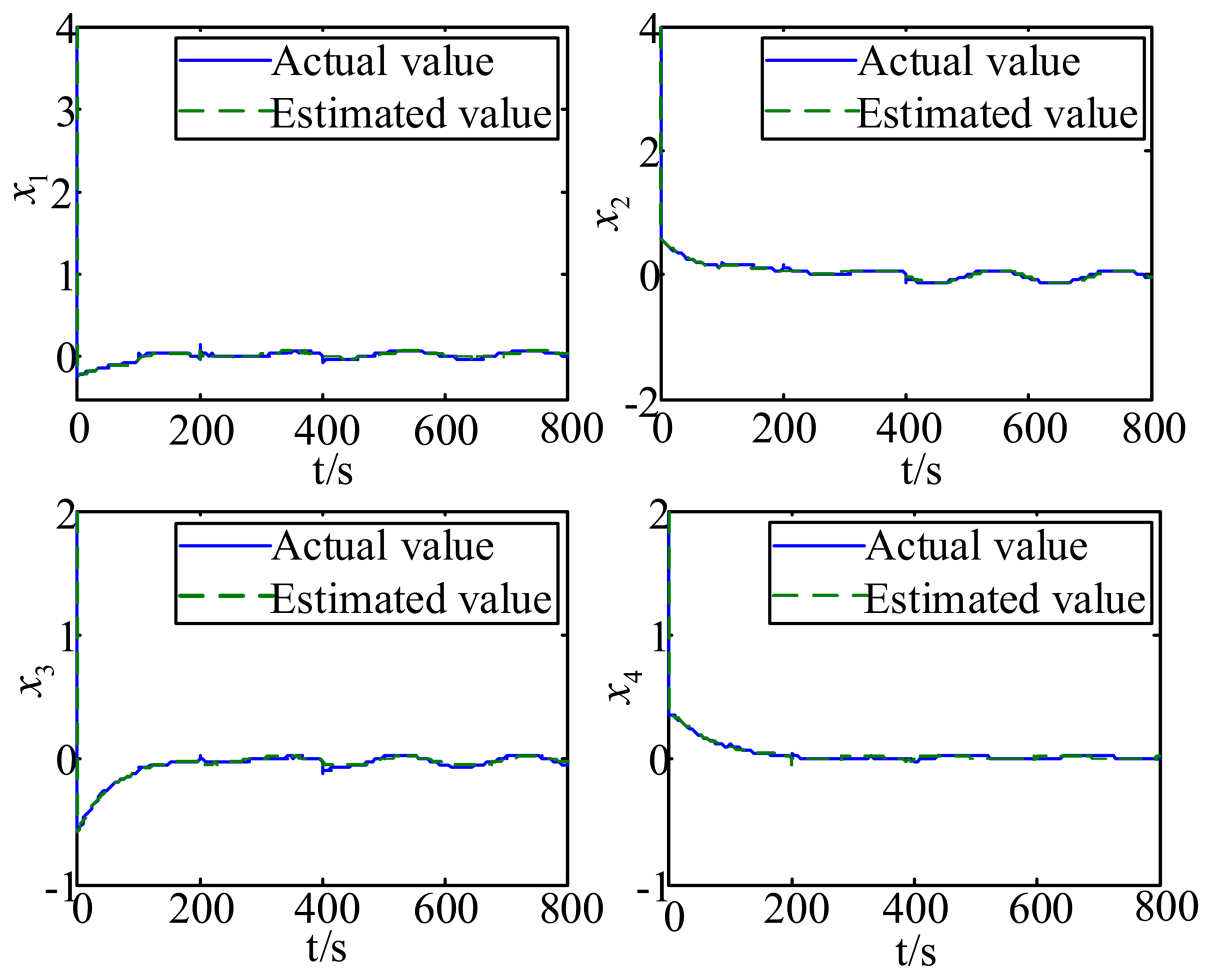
States and their estimation.

**Figure 9:**
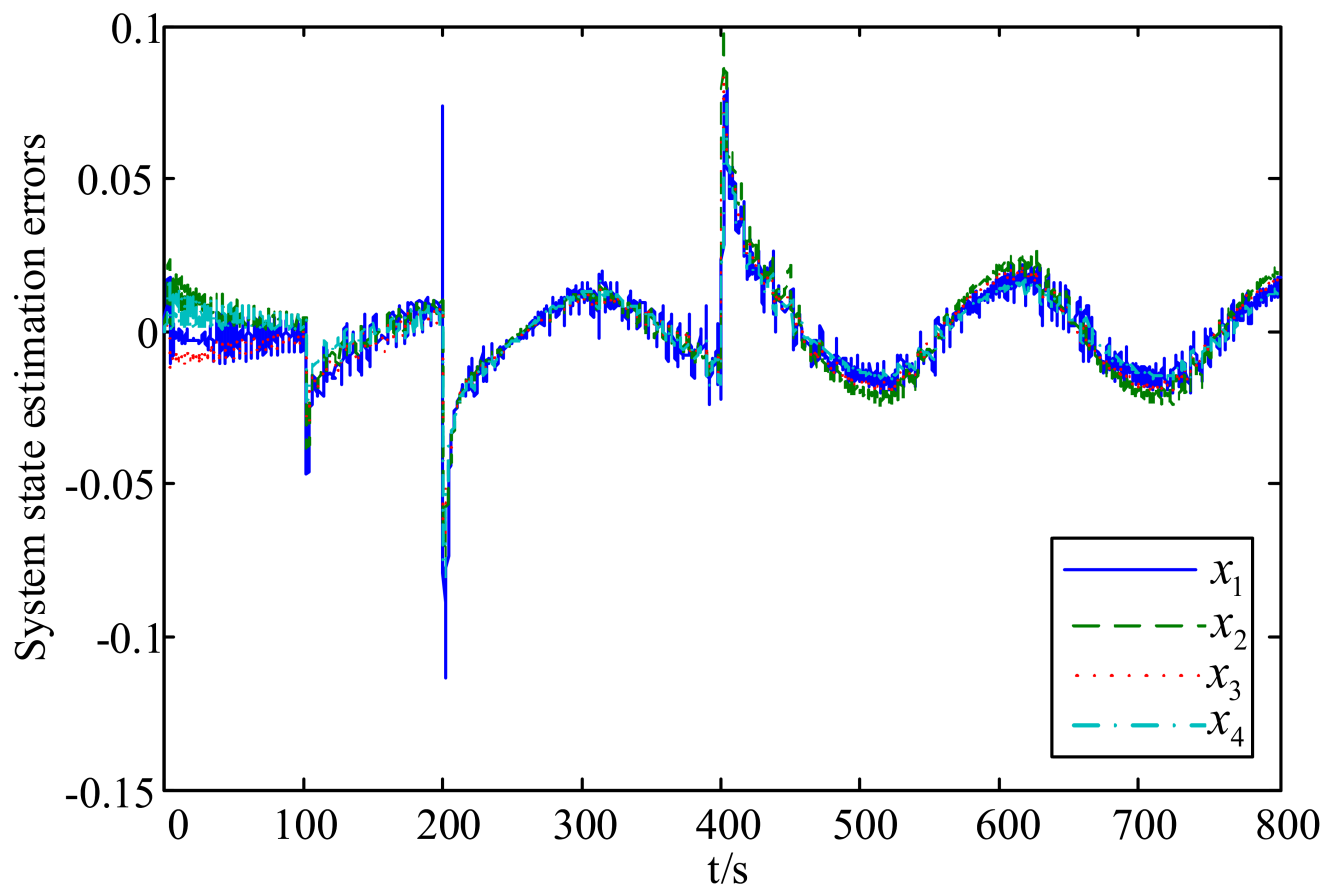
Estimation errors of system states.

Under the coordinated operation of the robust observer and the integrated secure controller, the system states, their estimates, and the state estimation errors are depicted in Figure 8 and Figure 9. 

As illustrated in Figure 8 and Figure 9, when t<100s, the system states rapidly converge to the steady state. During the interval 100s≤t<200s, an FDI attack is injected into the actuator-side network. The system response remains stable; a transient spike in the estimation error reaches −0.05 only at the attack onset, after which the estimated states closely track the actual values without significant oscillations. For 200s≤t<400s, dual-end (actuator and sensor) FDI attacks are activated. A minor fluctuation occurs at t=200s, with the maximum estimation error peaking at −0.1. The system subsequently maintains stability with negligible deviation. In the final phase (400s≤t≤800s), dual-end FDI attacks coexist with an actuator fault. Although a transient error spike of 0.1 appears at t=400s, the state fluctuations remain minimal, and the estimation error is consistently bounded within [−0.025,0.025], rendering its impact on closed-loop performance negligible.

To further demonstrate the superiority of the proposed framework, comparative simulations against existing representative strategies are conducted in what follows.

### 5.4. Comparative Analysis

#### 5.4.1. Control Performance Comparison

**Figure 10:**
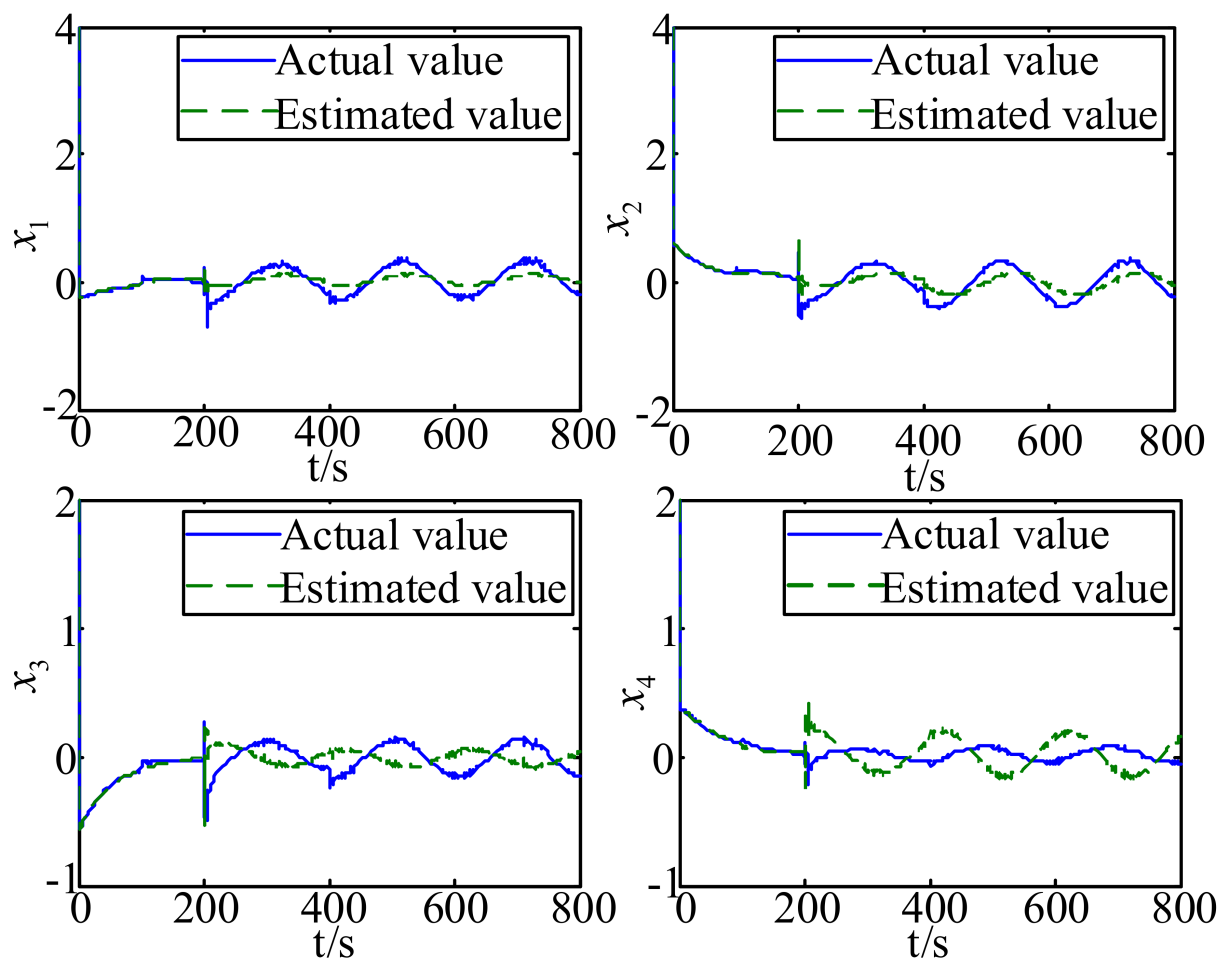
System states and their estimation with active–passive attack tolerance in [23].

**Figure 11:**
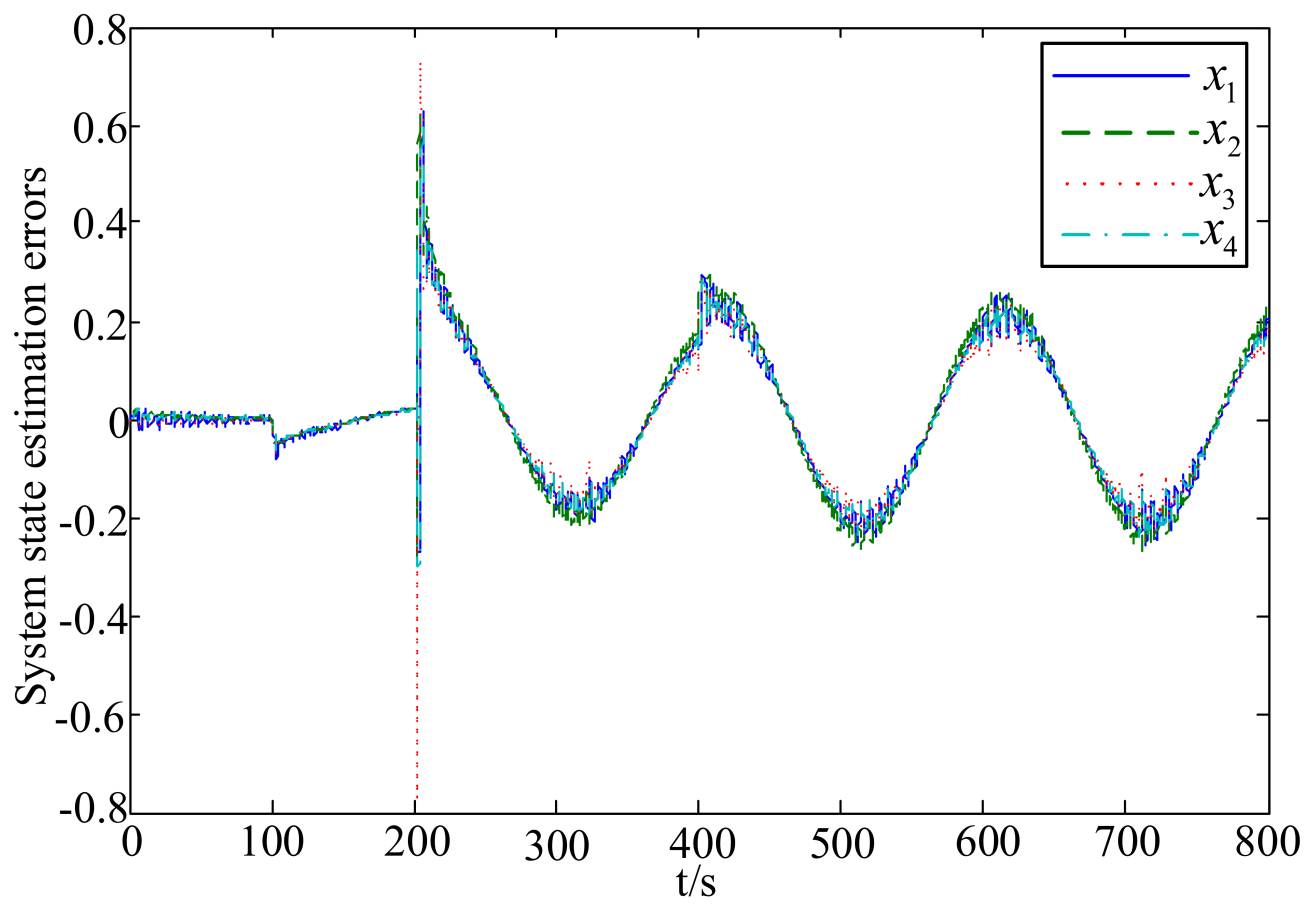
Estimation error of system states with active–passive attack tolerance in [23].

To further validate the effectiveness and advantages of the proposed active attack/fault-tolerant integrated secure control strategy based on ADETCS, a comparative simulation is conducted under identical operating conditions using the active–passive intrusion-tolerant and active fault-tolerant strategy reported in Ref. [23]. The resulting state responses are shown in Figure 10 and Figure 11. Analysis reveals that while the passive strategy maintains basic stability under dual-end FDI attacks, the system states in Figure 10 oscillate within ±0.5, and the state estimation errors in Figure 11 fluctuate within ±0.4. Compared to Figure 8 and Figure 9, these bounds are significantly larger, demonstrating that the proposed active intrusion/fault-tolerant control strategy delivers superior robustness and dynamic performance under concurrent dual-end FDI attacks and actuator fault.

While the above results confirm the advantages in control performance, the resource-saving capability of the proposed ADETCS remains to be quantified. The following subsection presents a comparative analysis of communication and computational overhead.

#### 5.4.2. Communication Resource Comparison

**Figure 12:**
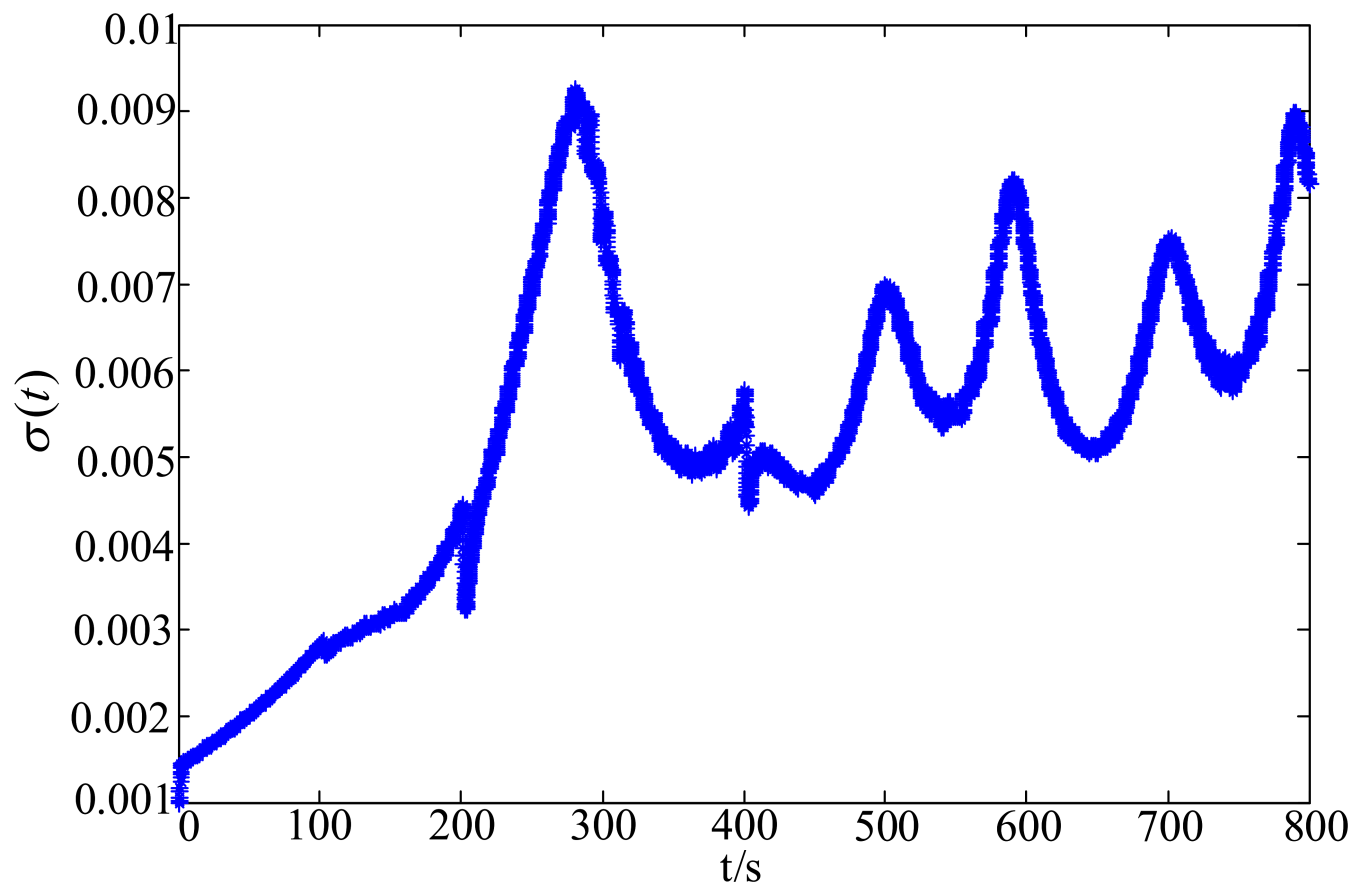
Adaptive event-triggered parameter variation diagram.

**Figure 13:**
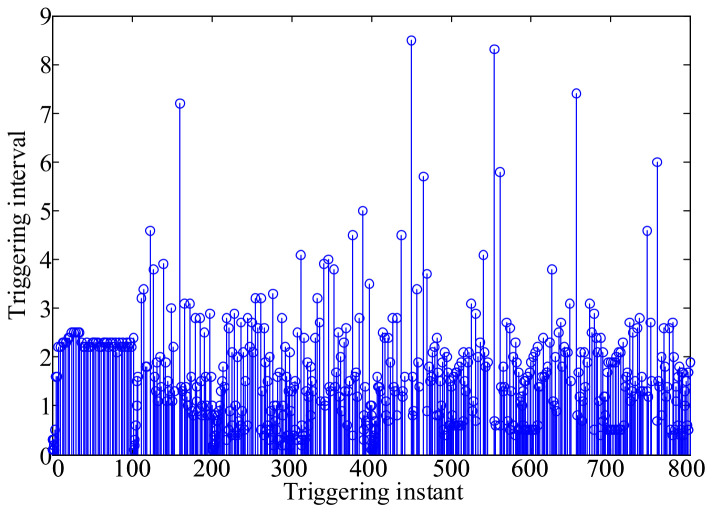
Triggering instants and inter-event intervals.

Figure 12 depicts the variation curves of the event-triggering parameters under the novel ADETCS proposed in this paper. As can be observed from Figure 12, the triggering parameters vary adaptively with the system dynamics, thereby reducing redundant data transmissions. Correspondingly, the triggering instants and inter-triggering intervals of the adaptive triggering parameters are shown in Figure 13.

Furthermore, to demonstrate the performance advantages of the proposed ADETCS, Table 3 lists the amount of data transmissions, data transmission rates, and data transmission periods under the active intrusion-tolerant strategy for the DETCS in [23], the ADETCS in [27], and the ADETCS proposed in this paper.

As shown in Table 1, the dynamically varying triggering parameters under the proposed ADETCS significantly reduce the data transmission rate. Over an 800-s simulation horizon, the number of transmitted data packets decreases from 1450 (required under the DETCS in [23]) to 712, with the corresponding transmission rate dropping from 18.1% to 8.9%. Compared with the ADETCS in [27], the proposed scheme introduces an upper bound on the triggering threshold, which indirectly constrains the maximum inter-event interval and thereby achieves a further reduction in data transmissions, offering superior conservation of network resources. Furthermore, since the robust observer is relocated to the control unit, estimation is performed only at transmission instants; this reconfiguration reduces the computational load at the sensor node from 8000 sampling-based calculations to merely 712 triggered executions over the 800-s interval. Consequently, the novel ADETCS developed in this paper achieves substantial savings in both network communication resources and system computational resources.

To summarize, the simulation results in this section conclusively demonstrate that the designed robust observer and integrated secure controller effectively achieve fault-tolerant control for actuator failure. Moreover, through their synergistic operation, the closed-loop system exhibits excellent intrusion tolerance against dual-end FDI attacks, validating the efficacy of the proposed co-design framework. Compared with existing strategies, the proposed scheme delivers superior robustness and dynamic performance while significantly reducing communication and computational burdens, thereby striking a favorable balance between security, performance, and resource utilization.

## 6. Conclusions

This study addresses integrated secure control and ADETCS co-design for nonlinear CPSs subject to coexisting FDI attacks and actuator faults. The main conclusions are as follows.

First, to tackle the low resource utilization of conventional triggering mechanisms, a novel adaptive discrete event-triggered communication control scheme (ADETCS) is proposed. By virtue of the dynamically self-adjusting triggering threshold that varies with the system state, the proposed mechanism achieves a significant reduction in network communication overhead while preserving the desired control performance.

Second, an integrated secure control framework that unifies computational resource allocation and communication optimization is constructed to enable an active defense strategy against dual-end FDI attacks. On this basis, a robust $H_{\infty }$ observer capable of real-time estimation of FDI attacks and actuator faults is designed, and an integrated secure control and communication co-design method is proposed for active intrusion tolerance against dual-end FDI attacks and active fault tolerance against actuator failures.

Finally, simulation results demonstrate that the proposed integrated secure control method exhibits a remarkable advantage over the passive attack-tolerant counterpart in actively counteracting FDI attacks and actuator faults. Meanwhile, the novel ADETCS outperforms the conventional DETCS in saving network communication resources, and the new system architecture effectively reduces computational resource consumption, thereby achieving a more favorable balance between performance and resource utilization.

Despite the promising results, several limitations should be acknowledged. First, the assumption of fully known system matrices restricts applicability in practical scenarios with model uncertainties. Second, the centralized observer architecture concentrates the estimation burden on the controller side; as the system dimension or the number of simultaneous attack/fault channels grows, the online computational load of the augmented observer may become non-negligible. Third, the validation is confined to simulation studies on a single benchmark system, and experimental verification on physical testbeds remains to be conducted.

Future work will focus on (1) relaxing the dependence on exact system parameters via adaptive or data-driven observer techniques; (2) extending the framework to distributed event-triggered security control for large-scale multi-agent CPSs under coordinated attacks; and (3) conducting experimental validation on physical testbeds to verify the real-time implementability of the proposed framework.

## Figures and Tables

**Table 1:** Comparison of representative related works.

Ref.	System	Communication Scheme	Attack Type	Fault Type	Core Method	Limitation
[16]	CPS	Continuous	Stealth, replay, FDI (unknown input)	—	Geometric model-based monitors	No ET; no fault; no FTC
[17]	CPS	Periodic/Time-triggered	Learning-based Man-in-the-Middle (MITM) attack	—	Variance/covariance detection; LS learning	No ET; no fault/FTC
[18]	CPS	Periodic/Time-triggered	FDI	—	KL divergence attack design; χ^2^ detection	No ET; no fault/FTC
[19]	CPS	Periodic/Time-triggered	FDI	—	Kalman-consensus filtering; constrained optimization	No ET; no fault/FTC
[20]	Linear large-scale interconnected systems	Periodic/Time-triggered	Covert man-in-the- middle attacks	—	Dual-observer residual-based detection	No ET; no fault/FTC
[21]	CPS	Periodic/Time-triggered	FDI	—	Blended detection combining watermarking and moving target	Detection only; No ET; no fault/FTC
[22]	NCS	Periodic/Time-triggered	FDI	—	Security metrics; dynamic output feedback synthesis; game-theoretic placement	No ET; no fault/FTC
[23]	Nonlinear CPS	Event-triggered (ET)	FDI	Actuator faults	Active–passive attack-tolerance + active fault-tolerance	Fixed ET threshold
[24]	Wind power CPS	Dynamic event-triggered	Additive attacks on sensors and actuators	Composite fault	Dynamic ET non-vulnerable FTC	Linearized model
[25]	DC microgrids	Dynamic event-triggered	Stealthy cyber-attacks	Actuator/sensor faults	Adaptive compensation mechanism	No FTC
[26]	CPS	ET (fixed threshold)	Random FDI	Actuator faults	Reduced-order fault estimator; fault-tolerant compensation controller	Fixed ET threshold
[27]	Power systems	ADETCS	—	—	Adaptive ET, *H*_∞_ load frequency control	No attack; no fault
[28]	RDCVNNs	Adaptive sampled-data-based event-triggering	—	—	ASDBETC; time-related LKF; LMI synchronization	No attack; no fault/FTC
[29]	Switched systems	ADETCS	FDI	—	Adaptive ET; delay system transformation	No fault/FTC
This study	Nonlinear CPS	ADETCS	FDI (sensor & actuator sides)	Actuator fault	Active attack-tolerance and fault-tolerance; co-design of control and communication	—

**Table 2:** List of Notations.

Symbol	Description
x(t)	System state vector
u(t)	Control input vector
f(t)	Continuous time-varying actuator fault
$a^{a}(t)$	Sensor-side FDI attack
$a^{s}(i_{k})$	Actuator-side FDI attack
w(t)	Exogenous disturbance
$y(i_{k}h)$	Sampled measurement output
$v(i_{k}h)$	Measurement noise
$\left\{i_{1}h\right.,i_{2}h,\cdots ,i_{k}h,\left.\cdots \right\}$	Sampling instants of the sampler
$\left\{t_{1}h\right.,t_{2}h,\cdots ,t_{k}h,\left.\cdots \right\}$	Triggering instants determined by the event generator
$\sigma (t_{k}h)$	Adaptive triggering parameter
Φ	Triggering weighting matrix
$L_{j}$	Observer gain matrix
$F_{j}$	Augmented fault estimation gain matrix
$K_{j}$	Integrated security controller gain matrix
$\hat{x}(i_{k}h)$	Estimated system state from the observer
$\hat{x}(t_{k}h)$	Latest state estimate satisfying the triggering condition and transmitted via the network

**Table 3:** Comparison of Data Transmission under Different Triggering Mechanisms.

Triggering Mechanism	Data Transmissions	Transmission Rate	Transmission Period (s)
DETCS in [23]	1450	18.1%	0.552 s
ADETCS in [27]	936	11.7%	0.85 s
ADETCS in this paper	712	8.9%	1.124 s

## Data Availability

Data is contained within the article.
